# A culturally adapted psychologically informed, audiologist-delivered, manualized intervention for chronic tinnitus patients in Malaysia: Protocol for a randomized controlled feasibility trial

**DOI:** 10.1371/journal.pone.0328348

**Published:** 2025-07-18

**Authors:** Mohamad Azmeer Sadali, Wan Syafira Ishak, Mahadir Ahmad, Derek J. Hoare

**Affiliations:** 1 Centre for Healthy Ageing & Wellness (H-CARE), Faculty of Health Sciences, Universiti Kebangsaan Malaysia, Kuala Lumpur, Malaysia; 2 Audiology Unit, Department of Otorhinolaryngology, Hospital Sungai Buloh, Ministry of Health, Selangor, Malaysia; 3 Audiology Program, Faculty of Health Sciences, Universiti Kebangsaan Malaysia, Kuala Lumpur, Malaysia; 4 Center for Community Health Studies (ReaCH), Faculty of Health Sciences, Universiti Kebangsaan Malaysia, Kuala Lumpur, Malaysia; 5 Clinical Psychology Program, Faculty of Health Sciences, Universiti Kebangsaan Malaysia, Kuala Lumpur, Malaysia; 6 National Institute for Health and Care Research (NIHR) Nottingham Biomedical Research Centre, Hearing Sciences, Mental Health and Clinical Neurosciences, University of Nottingham, Nottingham, United Kingdom; 7 Department of Speech and Hearing Sciences, University College Cork, Cork, Ireland; Universiti Malaya Fakulti Perubatan: University of Malaya Faculty of Medicine, MALAYSIA

## Abstract

**Background:**

Tinnitus, a phantom auditory perception, varies from mild awareness to intolerable sounds that can severely impact psychological well-being, sometimes leading to suicidal ideation. While its causality remains unclear, studies show a strong link between tinnitus severity and psychological distress. A manualized intervention developed in the United Kingdom for audiologists, using a low-intensity, psychologically informed approach, has been successfully trialed in Western populations. However, its feasibility in Asian settings, particularly in Malaysia with its diverse cultures, remains unknown.

**Methods:**

Phase I involves three steps: (a) translating the intervention manual into Malay, (b) systematically adapting its content for cultural relevance, and (c) piloting the intervention. Phase II is a 4-month randomized controlled feasibility trial involving chronic tinnitus patients (n = 20), stratified by severity and randomly assigned to either (a) the adapted intervention delivered by a trained provider or (b) standard care. Feasibility outcomes include a 10% recruitment rate among eligible patients, a 65% eligibility rate within the target population, and 80% retention. Primary outcomes assess tinnitus severity via the Tinnitus Functional Index, while secondary outcomes measure psychological distress using the Depression Anxiety Stress Scale-42. Assessments occur at baseline, post-intervention (1 month), and at a 3-month follow-up. At follow-up, semi-structured interviews guided by the Theoretical Framework of Acceptability will explore participants’ perceptions of the intervention.

**Discussion:**

This feasibility trial evaluates the practicality and acceptability of a culturally adapted, audiologist-delivered psychological intervention for chronic tinnitus patients in Malaysia. Unlike direct translations, it incorporates systematic cultural adaptation to ensure relevance for the target population. Findings will inform the viability of a future large-scale trial and the potential for expanding psychologically informed tinnitus care in audiology settings.

**Trial registration number:**

ACTRN12624001048572

## Introduction

Tinnitus is classified as chronic if it persists for more than 6 months. Chronic tinnitus has a global pooled prevalence of 9.8% in adults (range across studies from 4.7% to 19.3%), and is usually associated with occupational noise exposure and presbycusis [1]. The prevalence of chronic tinnitus in Malaysia is unknown, but 4.9% of adult patients receiving audiological rehabilitation at government hospitals are treated for tinnitus [2]. Individuals with similar psychoacoustic characteristics of tinnitus, such as similar timbre, pitch, loudness, and localization, do not necessarily experience the same level of severity and psychological distress affecting their lives [3]. Tinnitus severity can be explained by multifaceted tinnitus-related experiences encompassing perceived loudness, symptoms, functional impacts, and quality of life. On the other hand, tinnitus distress can be defined as the negative emotional states faced by individuals with tinnitus, not limited to tinnitus-related experiences alone [4].

Despite the lack of a definite cure, largely due to its unclear pathophysiology, there are currently two main approaches to managing tinnitus. The first is the biomedical approach, which focuses on treating underlying medical conditions that may cause tinnitus. This ranges from physicians prescribing medication for treatable conditions such as otitis media to surgeons performing procedures like the surgical removal of acoustic neuroma [5]. For tinnitus patients with sensorineural hearing loss, audiologists play a role by recommending acoustic-based therapies such as hearing aids and/or ear-level sound generators which can help reduce the perception of tinnitus loudness [5,6]. The second approach is the psychosocial approach, which addresses the psychological impacts of tinnitus. Psychoeducation about tinnitus reassures the individuals by emphasizing that the condition is a symptom of most common hearing difficulties, rather than a dangerous disease [7]. Psychological interventions such as cognitive behavioral therapy (CBT), typically delivered by psychologists, have shown strong evidence for promoting tinnitus tolerance [8]. Learning to identify and restructure negative thoughts, patients can improve their well-being, even if there is no subjective change in tinnitus perception [9]. Nevertheless, the biomedical approach is more commonly practiced worldwide, including in Malaysia, despite the mismatched concerns about tinnitus-related distress and appropriate interventions [10,11]. This preference is largely due to the standardized training provided during clinical degrees, which emphasizes physiological and pathological aspects of health. Consequently, healthcare professionals may be less equipped to consider the psychological and social dimensions of conditions like tinnitus. This mismatch between the needs of patients and the focus of clinical training can result in interventions that may not fully address the multifaceted nature of tinnitus distress.

Combining both approaches, the holistic concept of the biopsychosocial approach may seem far-fetched due to limited access to psychological services, which could impose unnecessary costs on patients. Additionally, the number of mental health specialists with adequate knowledge of tinnitus management is scarce [12,13]. Although psychologists are traditionally the primary and recommended providers of CBT for mental health issues, including tinnitus management, there is currently a paradigm shift in which healthcare professionals other than mental health specialists are being empowered to deliver low-intensity psychological interventions (LIPIs). This approach maximizes access to evidence-based treatments for individuals with mild-to-moderate mental health needs, using a minimum number of resources [14–17]. Particularly for the management of tinnitus, Burke & Refaie [18] in their recent scoping review discovered expanding evidence of audiologists providing CBT over the past decade. Nonetheless, it is worth noting that the majority of these studies were conducted in the United Kingdom (UK) or the United States of America (USA), highlighting a substantial research gap worldwide.

In the UK, the National Institute for Health and Care Excellence (NICE) [15] recommended research into the efficacy of psychological interventions delivered by appropriately trained healthcare professionals, such as audiologists, in their guidelines for tinnitus assessment and management. Addressing audiologists’ concerns about insufficient training and confidence in integrating psychological interventions into existing biomedical treatments, Taylor et al. [19] developed a psychologically informed, manualized intervention for tinnitus at the NIHR Nottingham Biomedical Research Centre. This low-intensity intervention is designed to guide audiologists in delivering relatively brief interventions, with specific guidance on frequency, pace, and session length [16,20,21]. In Malaysia, a 2022 report by the Ministry of Health indicated that patients receiving audiological services were ethnically diverse, with the majority being Malay (71%), followed by Chinese (14%), Indians (6%), and indigenous peoples from the Malaysian Peninsula and the Borneo states of Sabah and Sarawak (9%) [2]. Given Malaysia’s multicultural and multi-religious society, there is a pressing need to adapt the UK’s psychologically informed intervention to align with local cultural norms and religious beliefs. This adaptation is crucial to ensure the intervention is both respectful and acceptable within the Malaysian context. Once culturally adapted, the intervention’s efficacy and acceptability will need to be assessed among chronic tinnitus patients before it can be safely and effectively implemented by audiologists in Malaysia.

The eventual target of this research plan is to reduce tinnitus severity and distress among chronic tinnitus patients in Malaysia by incorporating standardized psychologically informed guidelines into the routine clinical management of tinnitus. This feasibility trial set out as a preliminary study with the following objectives:

1. To culturally adapt the UK psychologically informed, audiologist-delivered, manualized intervention for chronic tinnitus patients.
2. To explore the acceptability of the adapted intervention among chronic tinnitus patients in Malaysia.
3. To assess the feasibility of conducting a full-scale trial of the culturally adapted psychologically informed, audiologist-delivered, manualized intervention for chronic tinnitus patients in Malaysia.

## Methods and analysis

### Phase I: Adaptation phase

Referring to the World Health Organization (WHO) guideline on psychological interventions implementation manual: integrating evidence-based psychological interventions into existing services [22], this adaptation phase comprised three stages:

1. **Translation**

The original intervention package was translated into Malay, the target language. However, the manual, which is used by audiologists, remained in English, as proficiency in English is a prerequisite for their bachelor’s degree. Only the sample dialogues and scripts intended to guide consultations with patients were translated into Malay, while the accompanying toolkit of resources for chronic tinnitus patients was fully translated. Most translations were carried out by the research team during several committee meetings, allowing for collaborative decision-making and contextual sensitivity. Additionally, some resources were sent for professional translation to ensure high linguistic quality and cultural appropriateness. An expert read-through was performed by a bilingual tinnitus specialist and a mental health specialist to further refine the materials.

The translation of supporting tools, including patient handouts and questionnaires, followed a structured process based on WHO guidelines [23]:

- **Forward translation (English to Malay).** Initial translation was conducted by bilingual experts.
- **Expert review and harmonization**. Audiologists and language experts reviewed the translations for clarity and accuracy.
- **Back translation (if needed for validation) (Malay to English).** This step ensured that the meaning was retained.
- **Cognitive debriefing.** Testing was conducted with patients to confirm that the translated materials were comprehensible.
- **Final review and approval.** The adapted materials were refined based on feedback before finalization.

The Tinnitus Functional Index (TFI) is the only resource in this study used as an outcome measure, and a validated Malay version was used, as detailed in the Outcome Measures section. For all other translated materials, including the Tinnitus Sample Case History Questionnaire (TSCHQ), patient toolkit, and consultation scripts, the primary objective was to enhance linguistic accessibility and cultural relevance rather than to establish a psychometric measure. As such, these resources did not require formal validation. The translation process involved professional translation services for selected materials, followed by collaborative review and refinement by the research team. Additionally, permission to use copyrighted questionnaires, such as the TSCHQ and TFI, was obtained from the copyright owners in advance.

2. Adaptation

The cultural adaptation of the manualized intervention and resources was a critical step in ensuring their relevance and usability in the Malaysian context. This process aimed to address linguistic, cultural, and professional differences while preserving the original intent and effectiveness of the materials. A structured framework, adapted from Bernal et al. [24], was followed for adapting the manual for audiologists:

- **Review and identify key content.** Sections containing cultural references, idiomatic expressions, or examples that may not resonate with Malaysian audiologists were identified.
- **Modify cultural references**. Analogies, scenarios, and examples were adapted to be more familiar, incorporating local social situations, workplace norms, and culturally relevant contexts (e.g., replacing references to Western settings with Malaysian equivalents).
- **Ensure terminology consistency.** Professional terms commonly used by Malaysian audiologists were retained to maintain clarity and precision, avoiding oversimplifications that could alter meaning.
- **Pilot testing with audiologists.** A small group of audiologists reviewed the adapted content to ensure clarity, professional relevance, and cultural appropriateness.

Several structural and content adaptations were made to improve the usability of the manual. The content was reorganized into distinct sections and chapters for better navigation, and a flowchart was introduced to visually summarize the treatment process. Tabulation was employed for specific examples, such as addressing misconceptions about tinnitus, patient education responses, and behavioral modifications for bedtime routines and daily habits, making the information clearer and more accessible.

During adaptation, language nuances, idiomatic expressions, and culturally sensitive content were carefully modified. Sample dialogues and scripts were translated into simple, culturally appropriate spoken Malay. Complex English sentences were either simplified or removed to enhance accessibility for Malaysian audiologists, many of whom use English as a second or third language. Additionally, steps that were initially presented briefly were expanded into detailed, step-by-step guidance to support audiologists with varying levels of experience. Culturally inappropriate references, such as the phrase “go out to the pub” in the *Negative Thinking Example Formulation* resource, were replaced with culturally appropriate alternatives.

Following these adaptations, the manual and resources underwent validation through face and content validity assessments conducted by seven audiologists managing tinnitus patients across government and private sectors, each with a minimum of one year of experience. This evaluation assessed the comprehensibility and readability of the adapted materials to ensure their suitability for clinical use in Malaysia.

3. **Pilot testing**

Based on a suggestion from Hall et al. [25], at least eight chronic tinnitus patients are recommended for recruitment in a small-scale test of the adapted intervention. Ten patients were successfully recruited, using either English or Malay as the medium, during the recruitment period from 11/06/2024–13/09/2024. Of these, six participants completed the adapted intervention, while four dropped out. In addition to serving as a training period for the intervention provider, the piloted intervention involved the evaluation of efficacy and acceptability using similar validated questionnaires outlined in the Outcome Measures section. These insights were valuable for further refinements of the intervention.

### Phase II: Intervention phase

#### Feasibility trial design.

**Fig 1:**
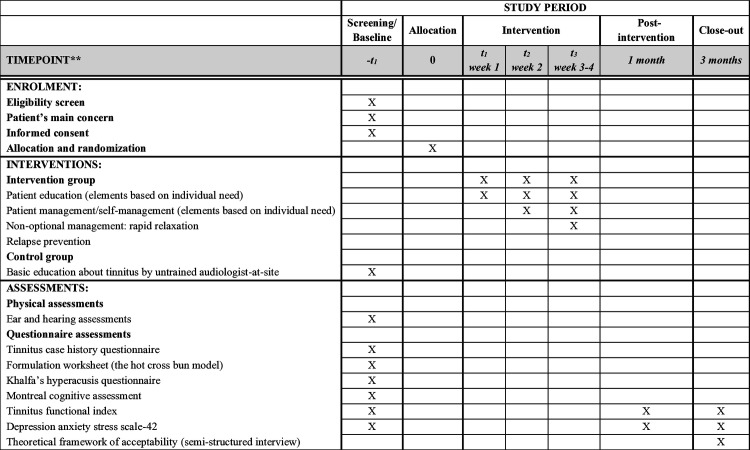
Schedule of enrolment, interventions, and assessments on participant outcomes, inspired by the SPIRIT 2013 reporting guidelines.

This study is a one-month randomized, double-blind (blinding both study participants and the outcome assessors), parallel-group (intervention and control groups) feasibility trial with a 3-month follow-up post-intervention. The overall schedule of the trial is shown in Fig 1. The study protocol was developed using the Standard Protocol Items: Recommendations for Interventional Trials (SPIRIT) [26] checklist as a guiding framework (see S1 Checklist). This trial is registered with the Australian New Zealand Clinical Trials Registry (trial registration number: ACTRN12624001048572). All items from the WHO Trial Registration Data Set are available in the trial registry entry. This randomized controlled feasibility trial began recruitment on 01/11/2024 and is currently ongoing. Participant recruitment is anticipated to be completed by 30/04/2025, with data collection expected to conclude by 31/07/2025. Study results are expected to be available by 30/09/2025.

### Sample size calculation

The sample size calculation was performed using G*Power software version 3.1 [27], with the effect size derived from a randomized clinical trial by Beukes et al. [28], which evaluated the effectiveness of guided internet-based cognitive behavioral therapy versus face-to-face clinical care in managing tinnitus. The Tinnitus Functional Index (TFI) was used as the primary outcome measure to assess tinnitus severity.

Using G*Power software under the F-test (analysis of variance, ANOVA) – repeated measures, within-between interaction (effect size f(v) = 0.45, alpha error probability = 0.05, power = 0.95, number of study groups = 2, number of measures = 3), the total sample size calculated is 16 participants. Considering a 10–20% dropout rate, a total of 20 participants will be recruited (10 participants per intervention group).

### Ethical approval and recruitment

The study protocol, written originally in English, was reviewed and approved by the Medical Research & Ethics Committees of the Ministry of Health, Malaysia (Ref: 23–03379-IUT) (see S1 Protocol) and Universiti Kebangsaan Malaysia (Ref: JEP-2024–420) (see S2 Protocol). Study participants will be recruited from adult patients with chronic tinnitus at the Otorhinolaryngology (ENT) Clinic, Hospital Sungai Buloh; the Audiology & Speech Sciences Clinic, Faculty of Health Sciences, Universiti Kebangsaan Malaysia (UKM); and the Audiology Unit, Department of Medical Rehabilitation Services, Hospital Canselor Tuanku Muhriz (HCTM) of UKM, based on eligibility criteria. They will be invited to voluntarily participate in the study, with appointments scheduled according to availability. Progress will be evaluated monthly to maintain the participant recruitment rate, allowing for a timely rectification of any issues in data collection. The study findings will be shared with enrolled participants during a debriefing session.

### Eligibility criteria

Inclusion criteria:

- Adults, aged ≥18 years, living in Malaysia
- Experiencing chronic subjective tinnitus for at least 6 months
- The ability to understand and write in simple Malay or English
- A tinnitus severity score of ≥25 on the Tinnitus Functional Index (TFI), indicating the need for intervention
• Completed a routine audiological assessment

Exclusion criteria:

- Tinnitus patients with a current history of outer and/or middle ear disorders
- Reporting pulsatile, objective, or unilateral tinnitus that has not been investigated medically or is still under medical investigation
- Undergoing any tinnitus therapy concurrent with participation in this study
- Having a diagnosis of mental health and/or neurological disorders
- Montreal Cognitive Assessment (MoCA) scores ≤ 25
- Tinnitus patients with coexisting hyperacusis, based on the patient’s self-report and Khalfa Hyperacusis Questionnaire (HQ) scores of ≥ 28.

Participants who meet all the inclusion and exclusion criteria will be briefed about the study, and written consent will be obtained by the researcher before they participate, along with an enclosed information sheet.

### Screening measures

At the screening stage, eligibility is determined based on a Tinnitus Functional Index (TFI) score of ≥25, along with additional measures:

- **Tinnitus Functional Index (TFI) (Self-administered):** The TFI is a widely used self-administered questionnaire for assessing tinnitus severity and will also serve as the primary outcome measure in this study. A baseline score of ≥25 is required for study inclusion to ensure participants experience a minimum level of tinnitus severity. Further details on its psychometric properties are provided under Outcome Measures.
- **Montreal Cognitive Assessment (MoCA) (Interviewer-administered):** The MoCA will be administered face-to-face by an interviewer at the screening stage to exclude participants with mild cognitive impairment, which could interfere with their ability to complete the intervention. It assesses multiple cognitive domains, including attention and concentration, executive functions, memory, language, visuo-constructional skills, conceptual thinking, calculations, and orientation [29] Similar to the English version, with passing scores of 26 and above, the Malay version of MoCA (MMoCA) exhibits a sensitivity of 0.824 and a specificity of 0.818 in identifying mild cognitive impairment [30].
- **Khalfa’s Hyperacusis Questionnaire (HQ) (Self-administered, English version only):** Hyperacusis is a decreased sound tolerance disorder that coexists in 30–50% of tinnitus patients, often leading to higher distress levels compared to those with tinnitus alone [31]. The HQ will be self-administered to assess participants’ tolerance toward noise. It consists of 14 self-administered questions, each rated on a 4-point Likert scale ranging from No (0) to Yes, a lot (3), with a maximum score of 42. Given that this study focuses primarily on tinnitus, participants who report additional issues with tolerating everyday sounds or have HQ scores ≥28, indicating strong auditory hypersensitivity, will be excluded [32]. As no validated Malay version of the HQ is currently available, the English version will be used.

### Interventions and randomization

To ensure a fair comparison, all 20 participants will first be stratified into two categories of tinnitus severity measured at baseline: small-to-moderate problems and large-to-very-large problems. The randomization sequence will be generated using a computer software program called Research Randomizer [33]. Participants within each severity category will then be randomly assigned into two groups (10 participants per group) by an independent research assistant who is not involved in participant enrollment or intervention delivery, ensuring allocation concealment. The randomization list will be securely stored, and group assignments will only be revealed once a participant has completed baseline assessments.

The two study groups are as follows:

1. *The intervention group* receiving the culturally adapted audiologist-delivered psychologically informed intervention
2. *The control group* receiving standard audiological care

Participants in both the intervention and control groups will receive their respective care at one of the three study sites, based on their recruitment location:

- Otorhinolaryngology (ENT) Clinic, Hospital Sungai Buloh
- Audiology & Speech Sciences Clinic, Faculty of Health Sciences, Universiti Kebangsaan Malaysia (UKM)
- Audiology Unit, Department of Medical Rehabilitation Services, Hospital Canselor Tuanku Muhriz (HCTM), UKM

#### Intervention group.

The adapted psychologically informed intervention will be delivered by the researcher to chronic tinnitus patients concurrently at three different study sites. The researcher is an audiologist with 10 years of clinical experience and has recently received specialized training in a dedicated workshop at University College Cork, Ireland, by the authors who developed the intervention manual. Based on a previous trial study, the intervention typically involved an average of 2.75 sessions delivered individually and face-to-face by an audiologist who attended a 2-day training workshop [19]. Therefore, in this study, each participant in the intervention group will receive three sessions (1 hour each), all of which are expected to be completed within 1 month.

Table 1 outlines the content of the intervention manual. The adapted intervention will be delivered mainly in the form of talking therapy, according to the participants’ preference of language, either in Malay or English. During Session 1, a patient assessment will be performed to determine which elements of patient education and patient management/self-management will be delivered, based on individual needs. At the end of the session, the participant will receive a treatment plan and relevant resources for reading and practice. In Session 2, further discussions will be based on the previously provided worksheets, using the toolkit of resources (Table 2). Additionally, participants will be encouraged to implement the non-optional module of rapid relaxation in patient management/self-management. Finally, in Session 3, after the participants have successfully managed the relevant elements, the intervention will be concluded with instruction on relapse prevention.

**Table 1:** Content of the culturally adapted psychologically informed intervention manual for chronic tinnitus patients.

Intervention Manual Content
**Section A. Introduction** Chapter 1. Background of the manual Chapter 2. Overview of the manual
**Section B. Patient assessment and preparation** Chapter 3. Patient assessment Chapter 4. Rationale for psychologically informed treatment Chapter 5. Goal setting/treatment planning Chapter 6. Patient education
**Section C. Intervention – Part I: Patient management/self-management** Chapter 7. Managing the emotional consequences of tinnitus Chapter 8. Rapid relaxation Chapter 9. Managing fear and avoidance behaviors Chapter 10. Changing unhelpful (negative) thoughts and beliefs Chapter 11. Promoting physical exercise Chapter 12. Promoting good sleeping habits Chapter 13. Attention, monitoring, and acceptance Chapter 14. Sound therapy (enrichment)
**Section C. Intervention – Part II: Concluding the intervention** Chapter 15. Relapse prevention
**Bibliography** **Appendices**

**Table 2:** Toolkit of resources available in both English and Malay.

Toolkit Resources
T1 - Tinnitus Functional Index (TFI)
T2 - Tinnitus Case History Questionnaire (TCHQ)
T3 - Formulation Worksheet (equivalent to the Hot Cross Bun Model)
T4 - BTA Leaflet: All About Tinnitus (Easy Read)
T5 - What Keeps Tinnitus Going (Cognitive Model of Tinnitus Distress)
T6 - Stages of Change Model
T7 - Goal Setting Sheet
T8 - Treatment Plan Sheet
T9 - Goal Setting and Action Planning Practice Framework
T10 - What Causes Tinnitus (from Malaysian Tinnitus Module, MyTiM)
T11 - Meaning in Tinnitus (from Psychology Tool)
T12 - BTA Leaflet: Tinnitus and Stress Ver.1.4
T13 - Relaxation Training Diary
T14 - Fear and Avoidance Example Formulation
T15 - Exposure Worksheet
T16 - Negative Thinking Example Formulation
T17 - Unhelpful Thinking Styles Sheet
T18 - Thought Record—Blank Copy
T19 - Thought Record—Example Copy
T20 - Physical Exercise Example Formulation
T21 - Physical Exercise Diary
T22 - Sleep Cycles Sheet
T23 - Sleep Diary
T24 - BTA Leaflet: Taming Tinnitus Ver.1.3
T25 - Acceptance in Metaphors Sheet
T26 - Mindfulness: Three Simple Ways to Get Present Sheet
T27 - Attention-Shifting Exercise Diary
T28 - My ‘Managing Tinnitus’ Blueprint: Making a Plan for Well-Being

#### Control group.

Participants in the control group who receive standard care will undergo a session of informational counseling about tinnitus (1 hour) following a routine hearing assessment. This session will be delivered by an experienced audiologist at the site who is not specially trained in managing tinnitus. The delivery procedures for both the intervention and control groups are detailed in a published protocol [34].

### Addressing potential performance bias

Both groups receive care across all three recruitment sites, ensuring consistency in clinical settings. The intervention group is delivered by an audiologist with ample clinical experience and specialized training in psychologically informed tinnitus management, while the control group is delivered by an audiologist with equivalent clinical experience but without specialized tinnitus management training. This difference is inherent to the study design, as the intervention is a specialized, psychologically informed approach that requires structured training, whereas standard audiological care does not involve psychological components. Since this mirrors real-world clinical practice in Malaysia, where a psychologically informed approach to tinnitus management is not widely adopted among audiologists, this study aims to assess its feasibility.

To minimize potential performance bias, the study employs randomization and allocation concealment to ensure that group assignment is not influenced by participant characteristics. Additionally, blinding of outcome assessors and the use of validated self-reported outcome measures further reduce the risk of bias in evaluating intervention effects.

### Blinding

Two groups of personnel are formed: an unblinded study team and a blinded study team. The unblinded study team consists of the researcher and research assistant 1, with research assistant 1 responsible for generating the random allocation and assigning participants to their respective groups. In contrast, the researcher who delivers the adapted psychologically informed intervention is responsible for conducting and monitoring the intervention, as well as scheduling sessions. The blinded study team consists of another two appointed research assistants who are unaware of the random assignments of the treatment groups. Research assistant 2 will provide self-administered questionnaires to participants at baseline, the end of the intervention, and 3-month follow-up. Meanwhile, research assistant 3 will analyse the outcomes at the end of data collection. As participants are also blinded to the type of treatments they are receiving, the randomized controlled trial process is considered double-blinded [35]. Theoretically, participants cannot distinguish between the experimental and control conditions, as both groups undergo routine audiological assessment and psychoeducation about tinnitus. Additionally, the type of intervention will not be disclosed during the debriefing session until participants have completed the study.

### Intervention fidelity

To ensure consistency in intervention delivery, the adapted intervention manual will be strictly followed. This structured manual provides a standardized framework for delivering the intervention across all sessions while allowing necessary clinical flexibility. The intervention provider will maintain a session log to document adherence to the manual and record any necessary modifications while ensuring that core intervention principles are upheld. This self-monitoring approach helps maintain fidelity without imposing unnecessary burdens.

Additionally, regular supervision meetings with the study supervisor will be conducted to review fidelity, address challenges encountered during sessions, and ensure continued alignment with the intervention framework. These discussions will serve as an additional fidelity check, providing opportunities for reflection and refinement as needed. By implementing these measures, the study aims to ensure that the intervention is delivered as intended, maintaining consistency and quality while allowing appropriate clinical adaptations when necessary.

### Outcome measures

To ascertain that future full-scale trials are up to the standards, running a feasibility trial beforehand is crucial so that efficacious interventions can be transferred successfully into the standard operating procedures [36]. Among the measures to confirm this feasibility are:

- A recruitment rate of 10% of all eligible candidates (recruiting one participant out of 10 chronic tinnitus patients screened).
- Recruitment of 65% of the target sample (13 participants out of 20 estimated sample size).
- A retention rate of 80% of participants adhering to the scheduled sessions parallel to the outcomes measured until the end of the study.

As for the efficacy of the intervention, the primary outcome measure relevant to tinnitus severity includes:

- **The Tinnitus Functional Index (TFI) (Self-administered).** This 25-item questionnaire provides a wider range of Likert scales compared to other available tinnitus questionnaires, as it is primarily developed to measure the effectiveness of tinnitus interventions. In the final development of TFI, Cronbach’s alpha was 0.97, and test-retest reliability was 0.78 [37]. The recently translated Malay version (TFI-Malay) also revealed good internal consistency, with a Cronbach alpha of 0.93 [38].

On the other hand, the secondary outcome measure used to evaluate tinnitus-related distress is:

- **The Depression Anxiety Stress Scale-42 (DASS-42) (Self-administered).** Despite the abbreviated 21-item DASS-21 being a more popular tool, the DASS-42 may provide higher reliability in scores, as well as more detailed information in explaining each psychological distress measured. The original DASS-42 has good internal reliability, with Cronbach’s alpha of 0.91 for depression, 0.81 for anxiety, and 0.89 for stress [39]. The translated Malay version has also shown good reliability with Cronbach’s alpha coefficient of 0.94, 0.90, and 0.87 for the respective domains [40].

Widely adopted in clinical settings for tinnitus management, these valid questionnaires will be administered at baseline, at the end of the intervention, and at the 3-month follow-up to detect therapeutic changes from the adapted intervention. Since the MoCA and HQ are also administered during the screening stage, we limit the number of questionnaires to reduce the risk of participant drop-out [41].

To further evaluate the intervention, acceptability will be assessed through semi-structured interviews guided by the Theoretical Framework of Acceptability (TFA) questionnaire. These interviews will be conducted physically or online, depending on participant preference.

- **The TFA questionnaire (Interview-administered, English version only):** The TFA was developed to provide a comprehensive framework for evaluating the acceptability of healthcare interventions and was later refined into a validated questionnaire (Sekhon et al.) [42,43]. It measures seven key constructs of acceptability: affective attitude, burden, perceived effectiveness, ethicality, intervention coherence, opportunity costs, and self-efficacy. The questionnaire has demonstrated good internal consistency across its constructs, with reported Cronbach’s alpha values ranging from 0.77 to 0.90 [43]. Participants will rate each construct on a scale from 1 to 5, accompanied by subjective comments to capture additional insights. As there is no validated Malay version of the TFA, the English version will be used.

### Assessment of outcomes and follow-up visits

Outcomes will be measured at baseline, post-intervention, and the 3-month follow-up through a combination of self-administered questionnaires and semi-structured interviews. Participants will have the option to complete self-administered questionnaires physically or online via Google Forms on the Linktree platform, depending on their preference.

The Tinnitus Functional Index (TFI) and the Depression Anxiety Stress Scale-42 (DASS-42) will be self-administered at all three time points to track changes in tinnitus severity and tinnitus-related distress. At the 3-month follow-up, acceptability will be assessed through semi-structured interviews guided by the Theoretical Framework of Acceptability (TFA) questionnaire, which will also be self-administered in English only. Interviews will be conducted physically or online, depending on participant preference.

**Fig 2:**
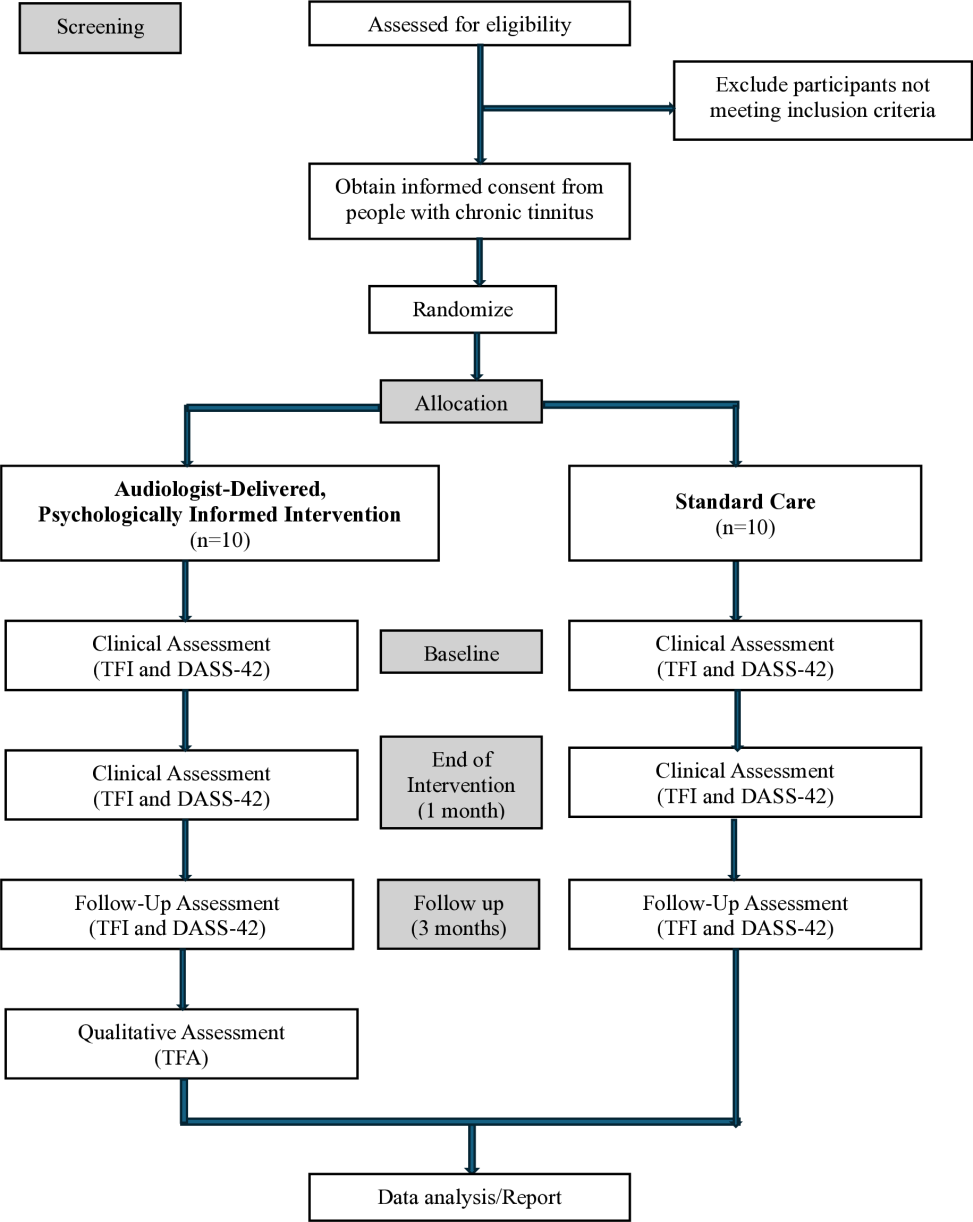
Study flow chart. TFI, Tinnitus Functional Index; DASS-42, Depression Anxiety Stress Scale-42; TFA, Theoretical Framework of Acceptability.

This study involves three face-to-face follow-up visits for the intervention group and one follow-up visit for the control group. The flow of the assessment in the study is illustrated in Fig 2.

### Assessment of compliance

To monitor participant adherence, the researcher will track the dates of the intervention sessions using a recorded form, updating the (re)scheduling of sessions as needed. The researcher will also contact participants via WhatsApp or phone call before their scheduled session as a reminder. However, participants may voluntarily withdraw from the study at any time.

### Adverse events

This study is conducted mainly in the form of talking therapy and psychoeducation, and all procedures are considered safe. Moreover, an adequate explanation of the procedures will be provided to patients verbally and literally. Breaks will be given as requested by the participant during procedures if needed. Though not anticipated, any adverse events will be reported to the principal investigator and the authority at the relevant study site.

### Amendments to the protocol

Any relevant amendments must be endorsed by the principal investigator and the researcher. If these amendments are significant and could affect participant safety or the efficacy of the trial, the Ethics Committee will be informed of the detailed changes. Approval from the research ethics committees of both the Ministry of Health and the university is required before implementing the amendments.

### Statistical analysis

An intention-to-treat analysis will be employed in the study, including data from all participants who were enrolled and intended to be treated, regardless of whether they completed the intervention. For participants with missing outcomes (dropped out or incomplete data), post-intervention scores will be imputed as pre-intervention scores, assuming the intervention does not worsen their tinnitus [44]. While collecting the participant data, all relevant sheets will be kept confidential and transferred to an online drive accessible only to the research members. IBM SPSS Statistics version 29.0.1.0 (171) will be used for all descriptive and inferential analyses.

The feasibility trial will not involve hypothesis testing, as its primary aim is to assess whether a full-scale trial is feasible. Descriptive statistics will be used to determine:

1. The recruitment rate of eligible participants at all sites.
2. The number of participants who consent to participate in the trial out of all eligible candidates.
3. The retention rate, i.e., the number of participants who complete the intervention and the 3-month follow-up.

For exploratory purposes and to inform future efficacy trial design, a mixed design ANOVA will be used to analyze changes in tinnitus severity and tinnitus-related distress across baseline, post-intervention, and the 3-month follow-up. This analysis incorporates both within-subjects (time) and between-subjects (group) factors, effectively capturing individual and group differences over time. The significance level will be set at *p* = 0.05. Effect sizes (partial eta squared, η²p) will be reported to quantify the magnitude of observed effects. Where applicable, corrections for multiple testing (e.g., Bonferroni adjustment) will be considered to control for inflation of Type I error.

To explore the acceptability of the adapted intervention among chronic tinnitus patients in Malaysia, ratings from the Theoretical Framework of Acceptability (TFA) questionnaire will be analyzed descriptively, while qualitative comments from semi-structured interviews will be analyzed using thematic analysis, following Braun and Clarke’s six-phase framework [45]. A deductive approach will be applied, with coding structured around the seven predefined TFA constructs. To ensure rigor and trustworthiness, two independent researchers will code the data, resolving discrepancies through discussion. Triangulation will be applied by comparing findings across TFA questionnaire ratings, interview responses, and researcher field notes, and member checking may be conducted to confirm the accuracy of participants’ perspectives. ATLAS.ti software will be used to facilitate data management, coding, and theme organization.

Post-trial, participants will be informed about the study results collectively with no reference to an individual and, where applicable, provided with referrals or guidance for continued care. If the study intervention proves to be effective, participants will be given information on how they might access it or equivalent treatments outside the trial. Other than participants, findings will be shared with other healthcare professionals and researchers through conferences and publications. A de-identified version of the dataset will be made openly available in a public repository following publication, in accordance with prior approval from the relevant institutional ethics committees. Researchers may also contact the corresponding author for any additional information or clarification.

## Discussion

### Potential impact and significance of the study

If proven feasible, the culturally adapted, audiologist-delivered psychological intervention has the potential to expand management options for chronic tinnitus patients in Malaysia. Unlike previous studies that primarily translated intervention materials, this study incorporates systematic cultural adaptation through expert reviews and participant feedback, ensuring that the intervention is not only linguistically but also culturally appropriate. This aligns with best practices in implementation science, which emphasize that successful intervention delivery requires more than direct translation.

By integrating psychological principles into audiology-led care, this study introduces an innovative, structured, and manualised approach that enables audiologists to tailor treatments to individual tinnitus patients. Standardised modules ensure consistent delivery of evidence-based care across clinics. Moreover, as a low-intensity psychological intervention designed for chronic tinnitus patients with mild-to-moderate psychological distress, this approach facilitates appropriate referral pathways—only cases with severe psychological symptoms are referred to psychiatrists or psychologists. This structured care pathway has the potential to improve cost-effectiveness in tinnitus management. Previous research has shown that psychological interventions can reduce annual healthcare costs by up to 20% [46], suggesting that this intervention could contribute to cost savings for both patients and the healthcare system.

### Strengths and limitations of the study

This study is a randomized, double-blind, active-controlled, parallel-group feasibility trial that aims to assess the practicality and acceptability of a culturally adapted, audiologist-delivered psychological intervention for chronic tinnitus in Malaysia. Unlike prior studies that primarily assessed the efficacy, this feasibility trial evaluates key implementation factors such as recruitment and retention rates, the appropriateness of study procedures, the suitability of outcome measures, and the integration of the intervention in clinical settings. By systematically assessing feasibility before progressing to a full-scale randomized controlled trial (RCT), this study provides critical data to inform future implementation.

A key strength of this study is that it addresses a research gap in Asia, where psychologically informed tinnitus interventions remain scarce. While Mindfulness-Based Cognitive Therapy (MBCT) has been trialed in Thailand [47] and Cognitive Behavioural Therapy (CBT) is included in Japan’s tinnitus management guidelines [48], research on audiologist-delivered, psychologically informed tinnitus care remains limited, particularly in low- and middle-income countries (LMICs). This study represents the first evaluation of such an approach in Malaysia, offering insights into its feasibility within a real-world audiology setting.

However, this study has limitations. The short follow-up period may not capture the long-term effects or potential side effects of the intervention, limiting the understanding of its sustained feasibility. Additionally, participant adherence to the intervention may vary, influencing the dosage or timing of engagement, which could affect outcome trends. Another limitation is that only one trained intervention provider is available to deliver the intervention across all study sites. While this ensures consistency in implementation, it may not fully reflect the scalability of the intervention if delivered by multiple providers in a real-world setting. Future research should explore the feasibility of training and scaling up audiologist-led psychological interventions across multiple clinics.

### Contribution to the audiology profession

In tinnitus management, audiologists typically assess and diagnose hearing loss, prescribe hearing aids, evaluate the negative impacts of tinnitus, and provide sound therapy with appropriate counseling. Given the scarcity of mental health professionals trained in tinnitus-specific CBT, this study highlights the potential for audiologists to deliver structured, manualized psychological interventions.

By equipping audiologists with appropriate competencies and providing structured supervision from mental health specialists, this approach expands their scope of practice while ensuring intervention fidelity. This study underscores the feasibility of integrating psychologically informed care into audiology settings, offering a sustainable and accessible solution for tinnitus patients in Malaysia and other LMICs where mental health services are limited.

## Supporting information

S1 Checklist SPIRIT 2013 reporting checklist for the clinical trial protocol. (DOCX)

S1 Protocol Study protocol approved by the Medical Research and Ethics Committee, Ministry of Health Malaysia. (PDF)

S1 File Inclusivity-in-global-research-questionnaire. (DOCX)
