# Supplementary material for: A culturally adapted psychologically informed, audiologist-delivered, manualized intervention for chronic tinnitus patients in Malaysia: Protocol for a randomized controlled feasibility trial
Source: PLoS One. 2025 Jul 18;20(7):e0328348. doi: 10.1371/journal.pone.0328348 (PMC12273995; doi:10.1371/journal.pone.0328348)
Supplement: S1 Protocol — (PDF) [file pone.0328348.s002.pdf]

RESEARCH PROPOSAL

EFFICACY OF THE ADAPTED VERSION OF AUDIOLOGIST-DELIVERED LOW INTENSITY PSYCHOLOGICAL INTERVENTION IN IMPROVING SEVERITY AND DISTRESS AMONG CHRONIC TINNITUS PATIENTS IN MALAYSIA

PREPARED BY:
MOHAMAD AZMEER BIN SADALI (P127323)
MASTER OF HEALTH SCIENCES (HEARING & SPEECH)
CENTRE FOR HEALTHY AGEING & WELLNESS (H-CARE)
FACULTY OF HEALTH SCIENCES
UNIVERSITI KEBANGSAAN MALAYSIA
KUALA LUMPUR

MAIN SUPERVISOR:
DR WAN SYAFIRA BINTI ISHAK
AUDIOLOGY PROGRAM
CENTRE FOR HEALTHY AGEING & WELLNESS (H-CARE)
FACULTY OF HEALTH SCIENCES
UNIVERSITI KEBANGSAAN MALAYSIA
KUALA LUMPUR

CO-SUPERVISOR:
ASSOC PROF DR MAHADIR BIN AHMAD
CLINICAL PSYCHOLOGY PROGRAM
CENTRE FOR COMMUNITY HEALTH STUDIES (ReaCH)
FACULTY OF HEALTH SCIENCES
UNIVERSITI KEBANGSAAN MALAYSIA
KUALA LUMPUR

CONTENT

Page

CHAPTER I: INTRODUCTION

1.1 Background 4

1.2 Problem Statement 6

1.3 Rationale of Study 6

1.4 Significance of Study 7

1.5 Conceptual Framework 8

1.6 Research Question 9

1.7 Research Objectives

1.7.1 Main Objective 9

1.7.2 Specific Objectives 9

1.8 Hypotheses 10

CHAPTER II: LITERATURE REVIEW

2.1 Principles of Cognitive Behavioural Therapy (CBT) in Tinnitus Management 11

2.2 Psychologist-Delivered CBT for Tinnitus: Highly Evidenced 12

2.3 Audiologist-Delivered CBT for Tinnitus: Limited Evidence 12

2.4 Features of Low Intensity Psychological Intervention by Audiologists 13

CHAPTER III: METHODOLOGY

3.1 Introduction 15

3.2 Phase I: Cultural Adaptation of Taylor's Audiologist-Delivered Low Intensity Psychological Intervention for Tinnitus 15

3.2.1 Research Design 15

3.2.2 Instrumentation 16

3.2.3 Translators 17

3.2.4 Research Procedures 17

3.3. Phase II: Feasibility Trial of the Adapted Version of Audiologist-Delivered Low Intensity Psychological Intervention for Tinnitus 19

3.3.1 Research Design 19

3.3.2 Research Location 19

3.3.3 Sampling Method 19

3.3.4 Blinding 20

3.3.5 Research Subject Criteria 20

3.3.6 Sample Size Calculation 21

3.3.7 Instrumentation 23

3.3.8 Research Procedures 25

3.4 Statistical Analysis 27

4.0 RESEARCH PROCEDURE FLOWCHARTS 28

5.0 GANTT CHART 30

6.0 REFERENCES 31

7.0 APPENDICES 38

CHAPTER I: INTRODUCTION

1.1 Background

Tinnitus can be defined as the phantom auditory perception in the absence of corresponding external stimulus. In medical history, the term tinnitus is derived from the Latin word *tinnire,* which means 'to ring' or 'a ringing' (Baguley et al. 2022). Tinnitus can be classified into different categories such as subjective or objective, pulsatile or non-pulsatile, tonal or non-tonal, and acute or chronic. Subjective tinnitus, which can only be heard by the sufferers, becomes chronic as it persists over a period of 6 months. This type of tinnitus is more prevalent in adults as compared to the paediatric population, ranging from 4.1% to 37.7% and is usually associated with age-related hearing loss as well as prolonged exposure to noise (Jarach et al., 2022).

The impact of tinnitus differs from an occasional awareness of noise in the ears or head, to an intolerable sound that leads some persons to contemplate suicide. People with similar psychoacoustic descriptions of tinnitus, including its timbre, pitch, loudness, and localization, do not necessarily share a similar level of severity and its psychological distress on their life (Norena, 2023). Tinnitus severity can be explained by multifaceted tinnitus-related experience including perceived loudness, symptoms, functional impact, and quality of life. Conversely, tinnitus distress is a construct that represents the emotional distress associated with, but not necessarily caused exclusively by, the tinnitus percept (Clarke et al., 2024). Pinto et al. (2014) performed a systematic review to study association between tinnitus and psychiatric disorders.

Most of the studies showed clear correlation between tinnitus severity and presence of anxiety and depression; despite its unestablished cause-and-effect relationship. Severe tinnitus can cause psychological distress, on the other hand the presence of anxiety and depression may decrease an individual's tolerance towards tinnitus, hence exacerbation of the symptoms (Oosterloo et al., 2021).

To date, there is no cure for tinnitus as its underlying mechanisms are yet to be clearly understood. However, there are currently two available approaches in the management of chronic tinnitus. First is the biomedical approach, treating medical problems that may cause tinnitus. This ranges from physicians prescribing medication in cases of medically treatable conditions such as otitis media, to surgeons performing surgical procedures such as removal of acoustic neuroma (Wu et al., 2018). A cross-sectional analysis of a national survey in the United States performed by Bhatt et al. (2016) revealed that most tinnitus sufferers seek medications despite inadequate evidence on its efficacy, especially in subjective tinnitus. Meanwhile, subjective tinnitus with presented hearing loss can be addressed by audiologists by offering acoustic treatments such as hearing aids or ear-level sound generators, which may further help in reducing its loudness perception (Tunkel et al., 2014).

The other management option is adopting a psychosocial approach, treating tinnitus-related psychological impacts. Education about tinnitus reassures tinnitus sufferers as it emphasises the condition itself as a symptom of most commonly hearing difficulties, rather than dangerous diseases (Han et al., 2021). Psychological interventions mainly cognitive behavioural therapy, which are usually conducted by psychologists, are the most leading evidence in promoting tinnitus tolerance. As tinnitus patients acquire skills to identify negative thoughts and restructure them into more helpful ones, this improves their well-being despite

subjectively no difference in the tinnitus perception (McKenna et al., 2014). However psychosocial approach is not commonly practised worldwide including in Malaysia, notwithstanding the concerning tinnitus-related distress (Husain et al., 2020).

1.2 Problem Statement

As the biomedical approach is more commonly practised in Malaysia, there seems to be a mismatch between appropriate interventions and actual patients' needs, especially when tinnitus sufferers are more psychosocially affected. As most patients with chief complaints of tinnitus are referred to otorhinolaryngology (ENT) and audiology clinics, audiologists as one of the primary healthcare providers in tinnitus management have raised concerns in lack of training and confidence in integrating psychological interventions into existing biomedical treatment (Hoare et al., 2015). On the other hand, limited access to psychological services may create unnecessary costs to patients, as well as attrition of mental health specialists with adequate knowledge in tinnitus may impede the holistic idea of biopsychosocial approach (Wan Suhailah et al., 2015).

1.3 Rationale of Study

There is currently a paradigm shift in healthcare in which clinicians other than mental health specialists are empowered to deliver low intensity psychological interventions (LIPIs). The aim of such interventions is to maximise access for people with lower intensity mental health needs to evidence-based treatments using a minimum number of resources, while those with greater severity are referred for more intensive interventions (McEvoy et al., 2020). Particularly in the United Kingdom, the committee of National Institute for Health and Care Excellence (NICE)

in their guideline for tinnitus assessment and management recommended research exploring efficacy of psychological intervention delivered by appropriately trained healthcare professionals besides psychologists, for instance, audiologists (NICE, 2020).

In the meantime, Taylor et al. (2020) at NIHR Nottingham Biomedical Research Centre has developed and trialled an audiologist-delivered low intensity psychological intervention for tinnitus, or in short, ADPIT. The guidance manual was a collective work between patients, audiologists, cognitive behavioural therapists, and research professionals intended to provide audiologists with a standard reference and supplementary materials to work with tinnitus sufferers in a more psychologically informed way. The feasibility trial revealed that the ADPIT intervention is acceptable to both patients and audiologists.

As the ADPIT intervention is applied to Western population, there is a requirement for the intervention manual to be first culturally adapted to Malaysian population by considering linguistic nuances, idiomatic expressions as well as reviewing any content that may be culturally insensitive or inappropriate. Taking an example of the Negative Thinking Formulation worksheet, the usage of 'go out to the pub' may not be locally appropriate, hence requiring further modifications to ensure respect and acceptance within Malaysian culture. Having the ADPIT intervention adapted, its efficacy and acceptability can later be assessed among chronic tinnitus patients in Malaysia.

1.4 Significance of Study

The audiologist-delivered low intensity psychological intervention is anticipated to expand the management options for chronic tinnitus patients, thus allowing audiologists to better tailor

treatments to individual cases in Malaysia. Using the standardised modules ensures that tinnitus patients receive a consistent level of care and access to evidence-based intervention regardless of the clinic they visit. As low intensity psychological intervention is delivered to chronic tinnitus patients with mild-to-moderate mental health needs, only cases screened with severe psychological symptoms are referred to psychiatrists and psychologists, which potentially leads to cost effectiveness in managing tinnitus patients. Furthermore, previous studies have shown that psychological treatment can reduce a person's annual physical healthcare costs by 20% (Layard, 2017). Thus, effective treatments may lead to cost savings for both patients and the healthcare system.

1.5 Conceptual Framework

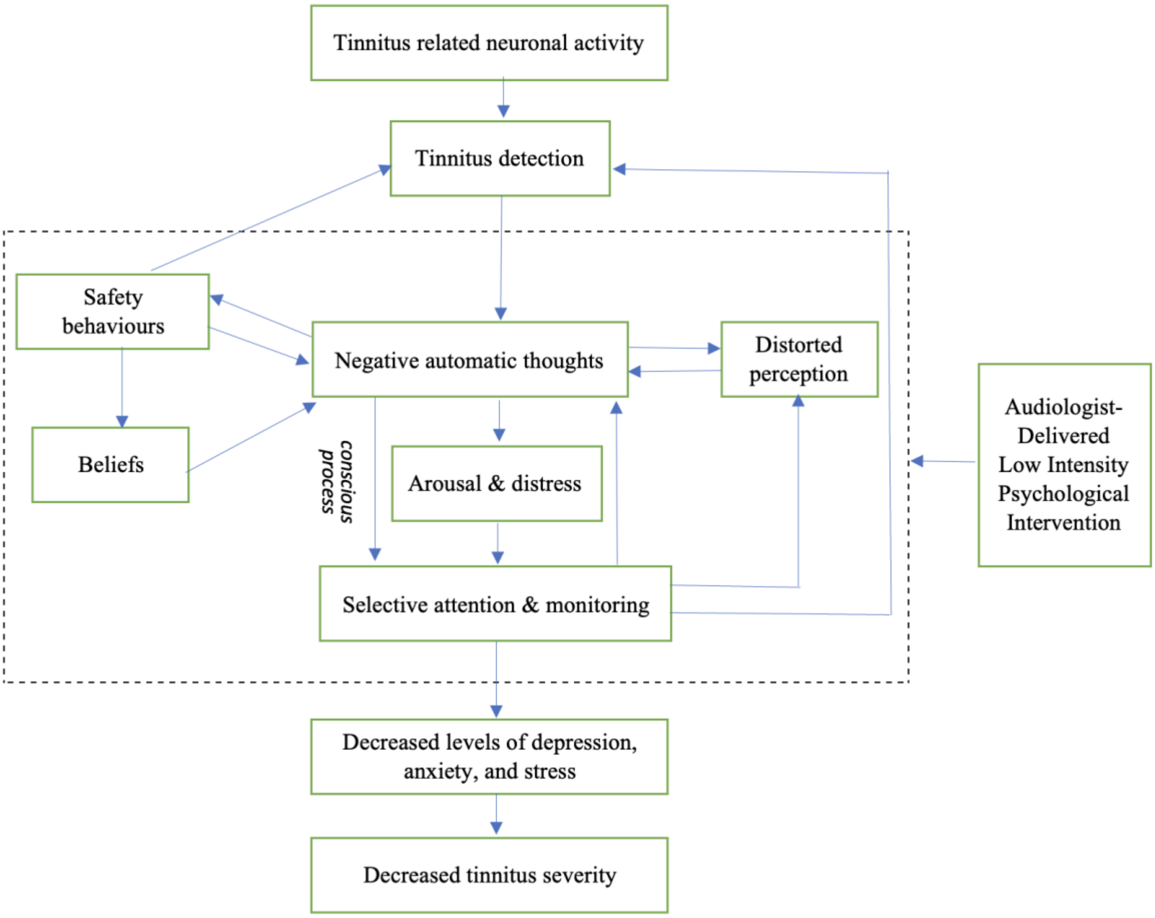

1.5 Research Questions

1. Is Taylor's audiologist-delivered low intensity psychological intervention (ADPIT) culturally appropriate?
2. Is there any significant reduction in tinnitus severity and tinnitus distress after receiving the ADPIT intervention?
3. Does the ADPIT intervention show efficacy in reducing tinnitus severity and tinnitus distress when compared to control group?
4. Is the adapted version of ADPIT intervention acceptable by chronic tinnitus patients in Malaysia?

1.6 Research Objectives

1.6.1 Main Objective

To evaluate the efficacy of audiologist-delivered low intensity psychological intervention in reducing tinnitus severity and tinnitus distress among chronic tinnitus patients in Malaysia.

1.6.2 Specific Objectives

1. To culturally adapt Taylor's audiologist-delivered low intensity psychological intervention for tinnitus (ADPIT).
2. To compare tinnitus severity and tinnitus distress between baseline, end of intervention, and 3-month follow-up post ADPIT intervention.

3. To assess the efficacy of ADPIT intervention by comparing tinnitus severity and tinnitus distress during baseline, end of intervention, and 3-month follow-up between treatment group and control group.
4. To assess the acceptability of ADPIT intervention among chronic tinnitus patients in Malaysia.

1.7 Hypothesis

1. There is a significant reduction in tinnitus severity and tinnitus distress after receiving the ADPIT intervention.
2. The treatment group shows significant reduction in tinnitus severity and tinnitus distress as compared to the control group.
3. The adapted version of ADPIT intervention is acceptable among chronic tinnitus patients in Malaysia.

CHAPTER II: LITERATURE REVIEW

2.1 Principles of Cognitive Behavioural Therapy (CBT) in Tinnitus Management

Psychological treatment considers cognitive processes to play a primary role in the perception of tinnitus. Originally used to treat depression, in the past years, a family of cognitive-behavioural models have been developed to account for chronic problems such as anxiety, pain and insomnia (Salkovskis et al., 1996; Sharp, 2001; Harvey, 2005). The models proposed that people experiencing these problems misinterpret their symptoms as evidence of serious physical illness. Distress persists as behavioural changes maintain the overly negative interpretations; and as these processes are motivated by inaccurate beliefs, an endless vicious cycle is created.

Similarly, regardless of the original cause of tinnitus, cognitive-behavioural processes contribute to the development and maintenance of distress starting with intrusive thoughts about tinnitus, overestimating its intensity and complexity. By performing CBT on these patients, behaviours which are cognitively mediated can be changed by a process of conscious cognitive enquiry (McKenna et al., 2014). Subsequently as less arousals occur, the idea of natural habituation to tinnitus, proposed by Hallam and colleagues in 1984, in which the sufferers get accustomed to tinnitus by rapidly losing its emotional meaning despite persistent stimulation can be achieved (Henry, 2023).

2.2 Psychologist-Delivered CBT for Tinnitus: Highly Evidenced

Efficacy of CBT in treating tinnitus by mental health professionals such as psychiatrists and psychologists appears to be reasonably established. One of the earliest reviews performed by Hoare et al. (2011), assessing 11 randomised controlled trials (RCT) examining CBT for tinnitus. Most of the studies reported significant improvements in tinnitus intrusiveness, with moderate effect size. Grewal et al. (2014) later also conducted a systematic review of eight studies assessing CBT relative to a no-treatment control group. They discovered that CBT resulted in significant improvement in both scores of quality of life and depression. Fuller et al. (2020) in their recent systematic review of 28 studies examining CBT on adults with tinnitus for more than 3 months, revealed that CBT may reduce impacts on quality of life at moderate level of evidence, when compared to inactive groups; and small effect size when compared to other active treatments. Nolan et al. (2020) on the other hand did a 10-year retrospective analysis by including 268 tinnitus patients who received CBT. The study revealed that CBT is highly effective in reducing tinnitus severity as well as accompanying distress.

2.3 Audiologist-Delivered CBT for Tinnitus: Limited Evidence

Limited studies have been conducted so far in determining the efficacy of CBT delivered by audiologists. Aazh & Moore (2018) conducted a retrospective study on 68 tinnitus patients with average age of 52.5 years who received audiologist-delivered CBT, where the results showed significant improvement of scores in tinnitus handicap, loudness, and annoyance after overall six sessions of behavioural experiment and diary of thoughts and feelings.

However, the study lacked a comparison group hence the placebo effect may have led to overestimation of its effectiveness. Taylor et al. (2020), after developing the ADPIT intervention manual which concentrated mostly on cognitive restructuring, performed an RCT to examine its efficacy and subsequently found a reduction in tinnitus severity, tinnitus cognitions and global distress at the end of intervention. An RCT was also performed recently by Beukes et al. (2022) who investigated the efficacy of 8 sessions of internet-based audiologist-delivered CBT, focusing mainly on mindfulness. They found greater reduction in tinnitus distress, negative tinnitus cognition and insomnia; and the results retained over the 2-month follow-up period. Nevertheless, more studies are required to explore potential benefits of internet-based therapy as compared to face-to-face therapy.

2.4 Features of Low Intensity Psychological Intervention by Audiologists

In providing care for tinnitus, audiologists assess and diagnose hearing loss, prescribe hearing aids, evaluate the negative impacts of tinnitus, and promote sound therapy with appropriate counselling when applicable. Since audiologists are already actively involved in tinnitus management, not to mention the scarcity of mental health specialists trained to implement CBT for tinnitus, there are arguments whether audiologists can perform psychological intervention (Henry et al., 2022). Various healthcare professionals such as physiotherapists, nurses, speech-language pathologists, and medical practitioners have practised psychological interventions and managed to improve clinical outcomes as well as patient wellbeing (Bryant et al., 2014; Sekhon et al., 2015; Liu et al., 2019; Morrell et al., 2014). Surprisingly, an audiologist named R. Sweetow was among the first to publish on implementation of a subset, rather than full CBT components in his tinnitus clinic (Sweetow, 1986). His version of CBT was designing

measurable strategies following identification of maladaptive behaviours and thoughts patterns associated with tinnitus.

Relevant to current situations, training for audiologists in delivering low intensity psychological interventions need not be as comprehensive as those received by psychologists. As long as competencies are met, audiologists may acquire supervision and oversight from mental health specialists (Bennett-Levy et al., 2010). Targeting tinnitus patients associated with less complex, mild-to-moderate mental health difficulties, audiologists may provide relatively brief interventions with respect to frequencies, pace, and length of sessions. (McEvoy at al., 2020). As opposed to formulation-driven interventions unique to individuals performed by psychologists, audiologists' scopes of practice in psychological interventions are more structured and customised to tinnitus, working within the limits of an intervention manual (Beukes et al., 2021). In addition to audiologist-delivered low intensity psychological intervention for tinnitus (ADPIT) is made highly accessible to both audiologists and tinnitus sufferers, its efficacy is also measurable as the utilisation of standard outcome measures is recommended to monitor improvement in tinnitus (Taylor et al., 2020).

CHAPTER III: METHODOLOGY

3.1 Introduction

The study is divided into two phases: 1) cultural adaptation of Taylor's low intensity psychological intervention for tinnitus (ADPIT), whereby the modules used at professional level are adapted culturally to Malaysian population, and the accompanying toolkit of resources for the use of chronic tinnitus sufferers are translated into Malay, and 2) feasibility trial of the newly adapted ADPIT intervention.

3.2 Phase I: Cultural Adaptation of Taylor's Audiologist-Delivered Low Intensity Psychological Intervention for Tinnitus (ADPIT)

3.2.1 Research Design

In phase I, the process of cultural adaptation of the ADPIT intervention will employ a cross-sectional design, referring mainly to a study by Hall et al. (2018) as they extensively delineated recommendations to translate and adapt hearing-related questionnaires for different languages and cultures.

3.2.2 Instrumentation

1. ***Taylor's Audiologist-Delivered Low Intensity Psychological Intervention for Tinnitus (ADPIT)***

   A psychologically informed guidance manual to support audiologists in the management of tinnitus patients. The 50-page manual comprises introduction, patient assessment, rationale for psychological intervention, goal setting, patient education, eight modules of patient management/self-management, and relapse prevention. Together with the manual, 28 toolkits of resources are available to facilitate the implementation of tinnitus management and support the patient's learning while navigating the process collaboratively with the audiologist (Taylor et al., 2020).

   Elements in patient education, delivered based on individual needs are causes and maintenance of tinnitus, misconceptions, information about the mechanism, its meaning, associations with hearing aids, hyperacusis, and noise abuse, habituation to tinnitus, relaxation training, things to avoid, taming tinnitus takes time, and about masking.

   The eight modules of patient management/self-management, which are also dependent on individual needs are managing the emotional consequences of tinnitus, rapid relaxation, managing fear and avoidance behaviours, changing unhelpful (negative) thoughts and beliefs, promoting physical exercise, promoting good sleeping habits, attention, monitoring and acceptance, and sound therapy (enrichment).

2. ***Comparability/Interpretability Rating Sheet***

   A standard questionnaire to measure the success of the translation process, in terms of similarity of words, phrases and sentences, as well as the degree to which the forward and back-translations bring about the same response despite different wordings. The questionnaire is in the form of Likert scales, ranging from 1 (extremely comparable/extremely similar) to 7 (not at all comparable/not at all similar) are used (Sperber, 2004).

3.2.3 Translators

At least two bilingual translators are required in the forward translation, one bilingual translator in the back translation, and another bilingual translator involved in the committee review. The four bilingual translators should be native speakers of the Malay language and hold certificates of excellent proficiency in locally accredited English tests such as Malaysian University English Test (MUET). Among the options are certified translators and Teaching of English as Second Language (TESL) educators.

3.2.4 Research Procedures

The process of cultural adaptation of Taylor's audiologist-delivered low intensity psychological intervention for tinnitus (ADPIT) starts with sufficient preparation especially by communicating with the original author to steer clear of violating copyright laws, to minimise the likelihood of misunderstanding during subsequent translation and adaptation phases, and to emphasise challenges encountered from previous experience. Only then two bilingual translators can perform translation from the source language (English) into the target language (Malay) independently, after the briefing on the intervention manual and translation

requirements. The modules need not be translated in view of professional usage by audiologists, who also hold competencies in English proficiency as a part of their bachelor's degree prerequisites. While the toolkits of resources are translated for the use of chronic tinnitus sufferers who come from different backgrounds of life.

Next, as the two independent forward translations are integrated, at least one bilingual translator translates the target language (Malay) back to the source language (English). The two-source language versions are then compared in a committee review consisting of at least an independent bilingual translator, an audiologist and a psychologist using the Comparability/Interpretability Rating Sheet. They should reach a consensus on any discrepancies to achieve cross-cultural equivalence in the forward translation. A pilot study with at least 8 chronic tinnitus patients is recommended to ensure that the intervention manual is comprehensible and culturally appropriate. There was no consensus from previous literature in specifying sample size for pilot study, but typically the range is between 5 to 50 (Hall et al., 2018). Subsequently based on the pilot data, the newly adapted intervention undergoes finalisation, including amendments, proofreading, the issuance of a translation certificate, and sharing with the original author before it is ready for circulation.

3.3 Phase II: Feasibility Trial of the Adapted Version of Audiologist-Delivered Low Intensity Psychological Intervention for Tinnitus (ADPIT)

3.3.1 Research Design

During phase II, the study will employ a mixed-method design, involving a feasibility trial to assess the efficacy of the adapted intervention quantitatively, in addition to a subsequent qualitative evaluation of its acceptability among chronic tinnitus patients via structured interviews.

3.3.2 Research Location

The study will be conducted at Otorhinolaryngology (ENT) Clinic at Hospital Sungai Buloh (government setting) and Audiology Clinic, Department of Medical Rehabilitation Services at Hospital Canselor Tuanku Mukhriz (university setting) as these specialised clinics are referral centres in receiving tinnitus cases from primary care centres.

3.3.3 Sampling Method

Stratified random sampling can be implemented to ensure an equal proportion of participants in tinnitus severity measured at baseline. Total sample size calculated will be divided into two categories: small-to-moderate problems and big-to-very big problems before further random assignments made by a computer software ‘Research Randomizer’ into both treatment and control groups.

3.3.4 Blinding

Two groups of personnel are formed: an unblinded study team and a blinded study team. The researcher who delivers the low intensity psychological intervention, is not blinded as he carries the responsibilities of performing and monitoring the intervention, as well as scheduling sessions. The blinded study team consists of 2 appointed research assistant who are blinded to the random assignments of the treatment groups. Research assistant 1 will be providing self-administered questionnaires to the participants to fill during baseline, end of intervention, and 3-month follow-up. Meanwhile research assistant 2 will be analysing the outcomes at the end of data collection. As participants are also blinded to the type of treatments they are receiving, the randomised controlled trial process is considered as double blinding (Eccleston et al., 2022). Theoretically, participants are unable to distinguish the experimental condition from the control, as both the intervention group and the control group undergo routine audiological assessment and educational counselling. Additionally, the type of intervention will not be disclosed during the debriefing session until participants have finished participating in the study.

3.3.5 Research Subject Criteria

Potential participants will be recruited among patients of Hospital Sungai Buloh and Hospital Canselor Tuanku Mukhriz based on eligible criteria. They will be contacted to join this study voluntarily and appointments will be made based on the patient's time availability. All subjects who are referred for tinnitus management within 1 February 2024 to 28 February 2026 are required to come to these clinics to participate in this study.

1. Inclusion Criteria

i. Adults, aged ≥18 years, living in Malaysia
ii. Suffer from chronic subjective tinnitus for at least 6 months
iii. The ability to understand and write in Malay or English
iv. A tinnitus severity score of ≥18 on the Tinnitus Functional Index (at least small problem) which indicates the need for an intervention
v. Completed routine audiological assessment
vi. Do not wear hearing aids or other amplification devices

2. Exclusion Criteria

i. Tinnitus patients with current history of outer and middle ear disorders
ii. Reporting pulsatile, objective, or unilateral tinnitus, which has not been investigated medically or still under medical investigation
iii. Undergoing any tinnitus therapy concurrent with participation in this study
iv. Formally diagnosed with mental health and/or neurological disorders
v. Montreal Cognitive Assessment (MoCA) scores ≤ 25 suggesting mild cognitive impairment

3.3.6 Sample Size Calculation

Referring to a meta-analysis study by Ma Y et al. (2021) to see the efficacy of CBT for insomnia in breast cancer patients, total sample size for both treatment and comparison groups is estimated to be 26 subjects. It is calculated using G*Power calculator (version 3.1) based on following parameters:

F tests - ANOVA: Repeated measures, within-between interaction

Analysis: A priori: Compute required sample size

Input:	Effect size f	=	0.335
	α err prob	=	0.05
	Power (1-β err prob)	=	0.95
	Number of groups	=	2
	Number of measurements	=	3
	Corr among rep measures	=	0.5
	Nonsphericity correction ε	=	1
Output:	Noncentrality parameter λ	=	17.5071000
	Critical F	=	3.1907273
	Numerator df	=	2.0000000
	Denominator df	=	48.0000000
	Total sample size	=	26
	Actual power	=	0.9612577

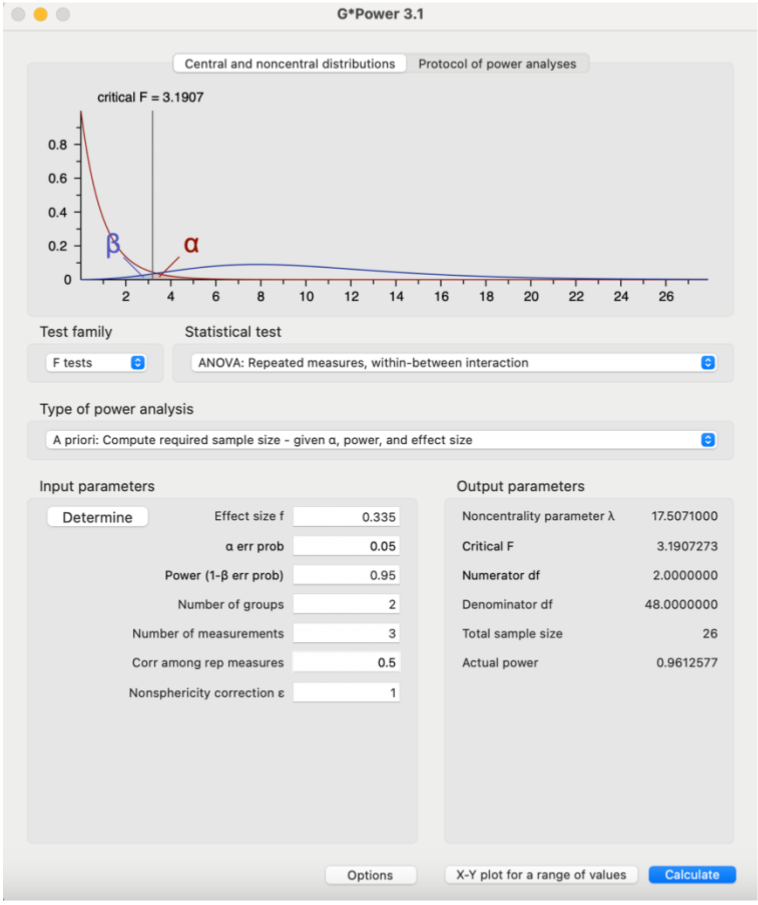

3.3.7 Instrumentation

1. ***Adapted Version of Audiologist-Delivered Low Intensity Psychological Intervention for Tinnitus (ADPIT)***

   The newly adapted ADPIT intervention contains similar modules and accompanying toolkits of resources, as explained in section 3.2.2.

2. ***Prosedur Operasi Standard Perkhidmatan Audiologi Dewasa (2023), Kementerian Kesihatan Malaysia***

   Participants are provided with basic information about tinnitus following complete standard care of audiological assessment.

3. ***Montreal Cognitive Assessment (MoCA)***

   Used at the screening stage to rule out potential participants with mild cognitive impairment that could interfere with carrying out the intervention. It assesses different cognitive domains: attention and concentration, executive functions, memory, language, visuo-constructional skills, conceptual thinking, calculations, and orientation. (Julayanont et al., 2013). With passing scores below 22, the Malay version of MoCA (MMoCA) exhibits a sensitivity of 0.824 and specificity of 0.818 in identifying cognitive impairment. (Kooi Cheah, 2014).

4. ***Visual Analog Scale (VAS)***

A self-rating scale for patients in determining level of loudness and annoyance with regards to their tinnitus, ranging from 0 to 10. This scale is included in the Tinnitus Functional Index.

5. ***Tinnitus Functional Index (TFI)***

A standardised questionnaire used to measure severity of tinnitus. The severity can be divided into "no problem" (score between 0 to 17), "small problem" (score between 18 to 31), "moderate problem" (score between 32 to 53), "big problem" (score between 54 to 72) and "very big problem" (score between 73 to 100). In the final development of TFI, Cronbach's alpha was 0.97 and test–retest reliability was 0.78. Convergent validity (r =0.86) with Tinnitus Handicap Inventory (THI); r=0.75 with Visual Analog Scale (VAS) and discriminant validity (r=0.56) with Beck Depression Inventory-Primary Care (BDI-PC) were good (Meikle et al., 2011). The recently translated Malay version (TFI-Malay) also revealed good internal consistency, with Cronbach alpha of 0.93. (Anis Aqila et al., 2023).

6. ***Depression Anxiety Stress Scale 42 (DASS-42)***

A self-report scale used to measure the negative emotional states of depression, anxiety, and stress. The severity levels are normal, mild, moderate, severe, and extremely severe; scored independently for each domain. The original DASS-42 has good internal reliability, with Cronbach's alpha of 0.91 for depression, 0.81 for anxiety, and 0.89 for stress (Lovibond & Lovibond 1995). The translated Malay version has also good reliability with Cronbach's alpha coefficient of 0.95. It also showed good concurrent validity with Hospital Anxiety Depression Scales (HADS) as the Spearman's

correlation values of 0.68 and 0.87 were obtained for depression and anxiety domain respectively (Ramli et al., 2012).

7. ***Theoretical Framework of Acceptability Questionnaire***

A structured questionnaire used to assess acceptability of an intervention. It consists of seven constructs: affective attitude, burden, perceived effectiveness, ethicality, intervention coherence, opportunity costs, and self-efficacy. For each construct, participants are asked to rate from 0 to 10 objectively as well as to provide subjective comments (Keyworth et al., 2021).

3.3.8 Research Procedures

Ethics approval will be obtained from Medical Research Ethics Committee (MREC) of Ministry of Health, Malaysia (KKM) and Research Ethics Committee of the National University of Malaysia (UKM).

After undergoing specialised training in a dedicated workshop at University College Cork, Ireland, the researcher who is an experienced audiologist will implement the low intensity psychological intervention and standard audiological care at two different study sites. Based on the previous trial study, the intervention involves an average of 2.75 sessions delivered one-to-one by an audiologist who attended a two-day training workshop (Taylor et al., 2020). Therefore, in this study, 3 sessions (1 hour each) are fixed for each participant in the intervention group and the control group. All three sessions are expected to complete within a month and reschedules of sessions are made whenever necessary.

The adapted intervention will be delivered according to the language preference of the participants, either in Malay or English. During session 1, patient assessment is performed to recommend which elements of patient education and patient management/self-management will be delivered, depending on individual needs. In session 2, further discussions are made based on the previously provided homework using the toolkit of resources. In addition, participants are also suggested to implement the non-optional module of rapid relaxation in patient management/self-management. Finally, during session 3, having coped with relevant elements, the intervention is concluded with relapse prevention. On the other hand, participants in the control group who receive standard audiological care undergo 3 sessions of informational counselling about tinnitus.

A total of 26 patients will be randomly assigned to one of two groups: (1) treatment group receiving audiologist-delivered low intensity psychological intervention for tinnitus (ADPIT) or (2) control group receiving standard audiological care. Quantitative outcome measures, including VAS, TFI and DASS-42 will be administered during baseline, discharge, and 3-month follow-up to determine the efficacy of the intervention. Intention-to-treat analysis is adopted in the study whereby data for all participants who enrolled for the ADPIT intervention who intended to treat, were included, regardless of whether they finished the intervention. For participants with missing outcomes (dropped out/incomplete data), post-intervention scores are set similar with pre-intervention scores, assuming the intervention does not worsen their tinnitus (Aazh et al., 2018). In addition, qualitative interviews will be conducted with the participants after 3-month follow-up to gather insights into the acceptability of the intervention. Ethically, if the intervention is proven its efficacy, the participants in the control group will be offered similar intervention in the treatment group following the analysis.

3.4 Statistical Analysis

1. Repeated measures ANOVA can be adopted to compare the tinnitus severity and the levels of depression, anxiety, and stress between baseline, end of intervention, and 3-month follow-up post ADPIT intervention.
2. Mixed design ANOVA will be used to assess the efficacy of ADPIT intervention by comparing the tinnitus severity and the levels of depression, anxiety, and stress at baseline, end of intervention, and 3-month follow-up between treatment group and comparison group.
3. Thematic analysis is suitable to assess the acceptability of ADPIT intervention among chronic tinnitus patients in Malaysia.

4.0 RESEARCH PROCEDURE FLOWCHARTS

Phase 1: Cultural adaptation of Taylor's audiologist-delivered psychological intervention for tinnitus (ADPIT)

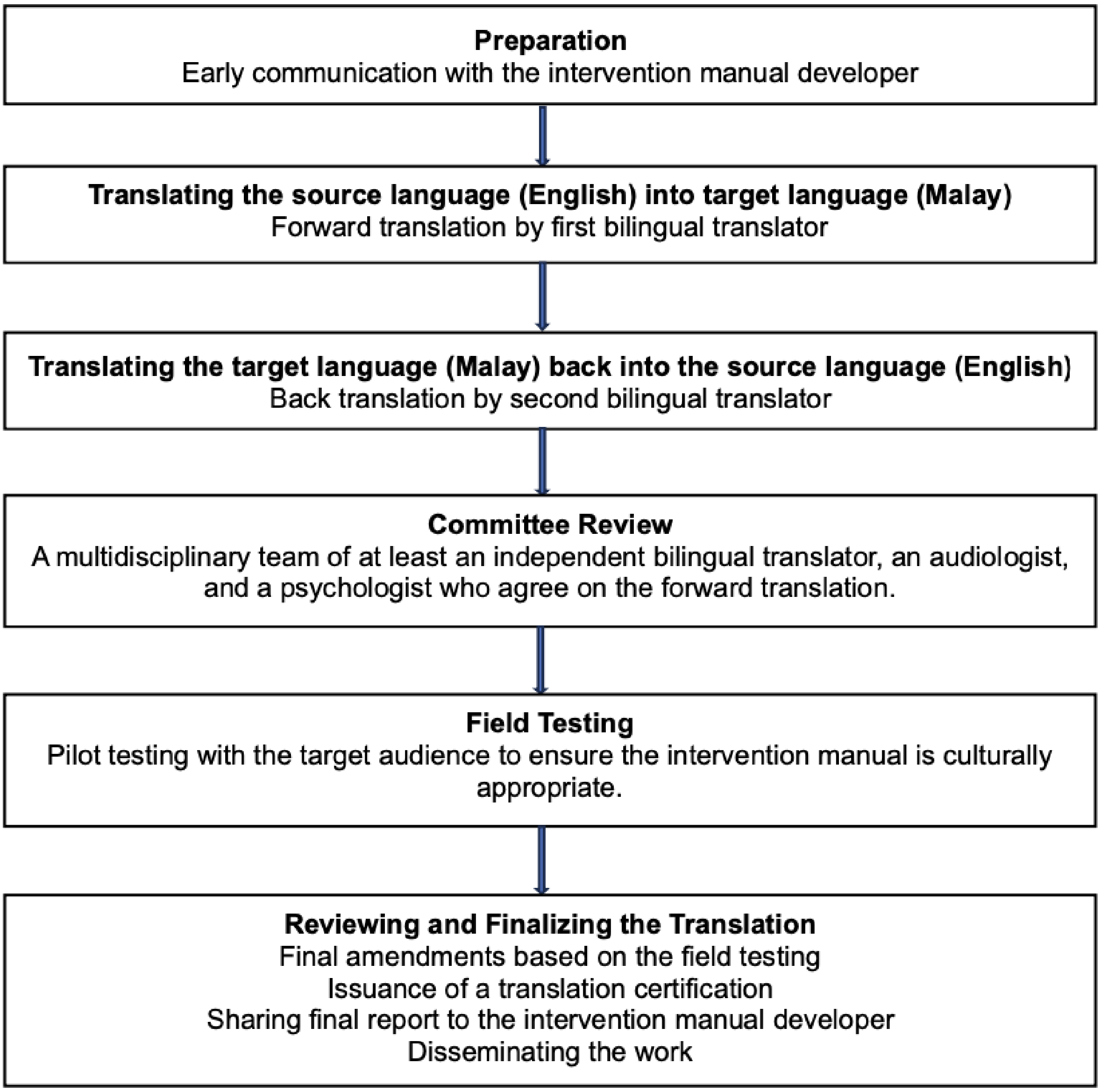

Phase 2: Feasibility trial of the adapted version of audiologist-delivered psychological intervention for tinnitus (ADPIT)

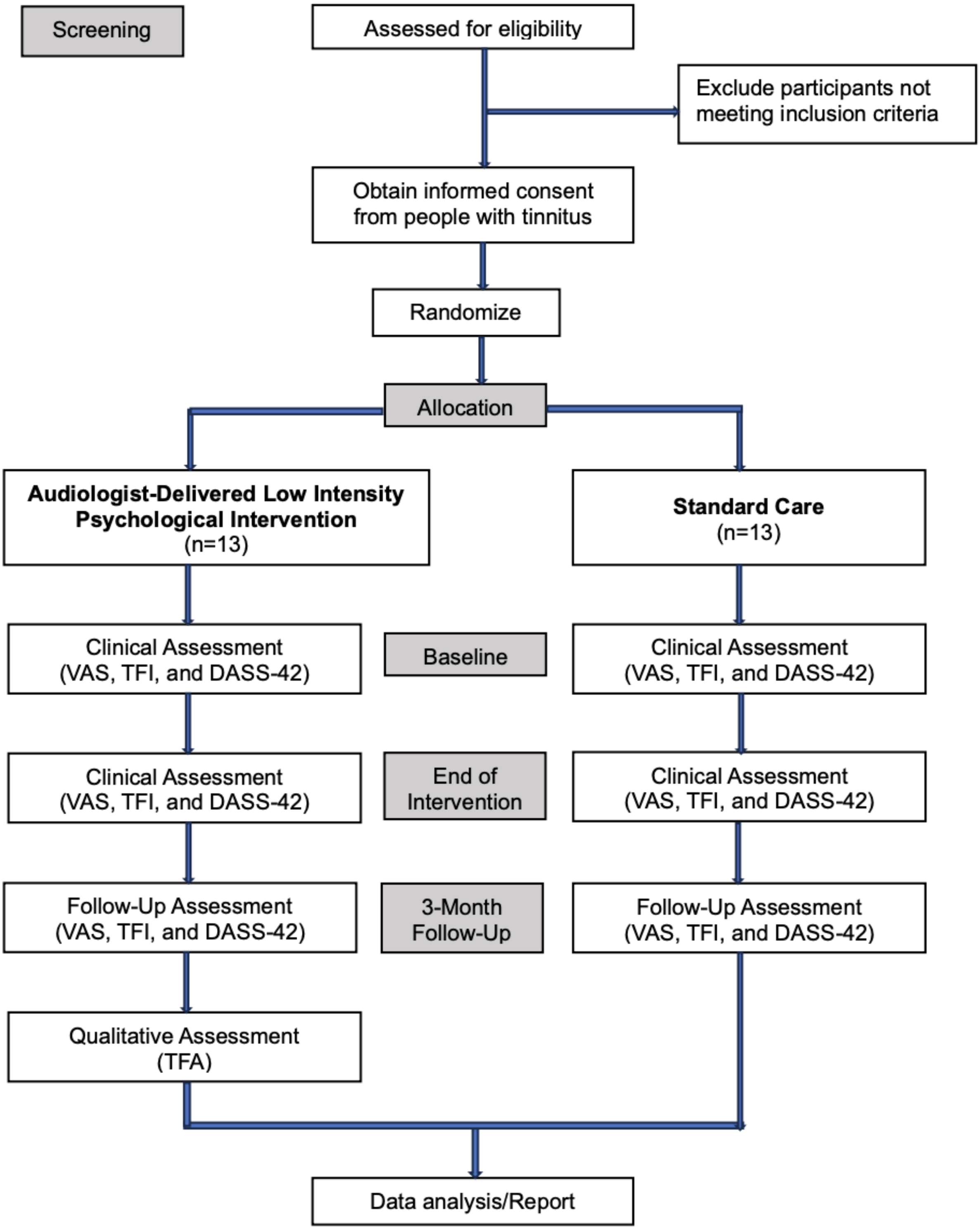

5.0 GANTT CHART

Research Activity	2023 Oct Dec	2024 Jan Mar	2024 Apr Jun	2024 Jul Sep	2024 Oct Dec	2025 Jan Mar	2025 Apr Jun	2025 Jul Sep	2025 Oct
Literature Review	■	■	■	■	■	■	■	■	■
Proposal Presentation		■							
Ethics Approval	■	■							
Data Collection		■	■	■	■				
Data Analysis		■	■	■	■	■	■		
Thesis Writing		■	■	■	■	■	■	■	
Final Report Submission							■	■	
Thesis Presentation									■

6.0 REFERENCES

Aazh, H. & Moore, B.C.J. 2018. Effectiveness of audiologist-delivered cognitive behavioral therapy for tinnitus and hyperacusis rehabilitation: Outcomes for patients treated in routine practice. *American Journal of Audiology* 27(4): 547–558.

Anis Aqila, A., Wan Syafira, I., Foong Yen, C. & Akmaliza, A. 2023. Validity and Reliability Of Tinnitus Functional Index (TFI) To Malay Language: A Preliminary Study. *Kolokium Prasiswazah Audiologi 2023* 33.

Baguley, D.M., Caimino, C., Gilles, A. & Jacquemin, L. 2022. The International Vocabulary of Tinnitus. *Frontiers in Neuroscience* 16(May): 1–6.

Bennett-Levy, J., Richards, D., Farrand, P., Christensen, H., Griffiths, K., Kavanagh, D., Klein, B., Lau, M.A., Proudfoot, J., Ritterband, L., White, J. & Williams, C. (Eds.). 2010, May 1. Oxford Guide to Low Intensity CBT Interventions. Oxford University Press.

Beukes, E.W., Andersson, G., Fagelson, M. & Manchaiah, V. 2022. Internet-Based Audiologist-Guided Cognitive Behavioral Therapy for Tinnitus: Randomized Controlled Trial. *Journal of Medical Internet Research* 24(2).

Beukes, E.W., Andersson, G., Manchaiah, V. & Kaldo, V. 2021. *Cognitive Behavioral Therapy for Tinnitus*. Plural Publishing, Inc.

Bhatt, J.M., Lin, H.W. & Bhattacharyya, N. 2016. Tinnitus Epidemiology: Prevalence, Severity, Exposures and Treatment Patterns in The United States: Bhatt JM: Tinnitus

in the United States HHS Public Access. *JAMA Otolaryngol Head Neck Surg* 142(10): 959–965.

Bryant, C., Lewis, P., Bennell, K.L., Ahamed, Y., Crough, D., Jull, G.A., Kenardy, J., Nicholas, M.K. & Keefe, F.J. 2014. Can physical therapists deliver a pain coping skills program? an examination of training processes and outcomes. *Physical Therapy* 94(10): 1443–1454.

Clarke, N.A., Hoare, D.J. & Trigg, A. 2024. A critical review of established tinnitus patient-reported outcomes as measures of Tinnitus Severity and Tinnitus Distress and exemplar analysis of the Tinnitus Handicap Inventory as a formative or reflective measure. Frontiers in Audiology and Otology 1.

Eccleston, C., Fisher, E., Liikkanen, S., Sarapohja, T., Stenfors, C., Jääskeläinen, S.K., Rice, A.S.C., Mattila, L., Blom, T. & Bratty, J.R. 2022. A prospective, double-blind, pilot, randomized, controlled trial of an "embodied" virtual reality intervention for adults with low back pain. *Pain* 163(9): 1700–1715.

Fuller, T., Cima, R., Langguth, B., Mazurek, B., Vlaeyen, J.W.S. & Hoare, D.J. 2020. Cognitive behavioural therapy for tinnitus. *Cochrane Database of Systematic Reviews* 2020(1).

Grewal, R., Spielmann, P.M., Jones, S.E.M. & Hussain, S.S.M. 2014. Clinical efficacy of tinnitus retraining therapy and cognitive behavioural therapy in the treatment of subjective tinnitus: A systematic review. *Journal of Laryngology and Otology* 128(12): 1028–1033.

Hall, D.A., Zaragoza Domingo, S., Hamdache, L.Z., Manchaiah, V., Thammaiah, S., Evans, C. & Wong, L.L.N. 2018. A good practice guide for translating and adapting

hearing-related questionnaires for different languages and cultures. *International Journal of Audiology* 57(3): 161–175.

Han, B.I., Lee, H.W., Ryu, S. & Kim, J.S. 2021. Tinnitus update. *Journal of Clinical Neurology (Korea)*.

Harvey, A.G. 2005. A cognitive theory and therapy for chronic insomnia. *Journal of Cognitive Psychotherapy* 19(1): 41–59.

Henry, J.A. 2023. Directed Attention and Habituation: Two Concept Critical to Tinnitus Management. *American Journal of Audiology* 32(2): 274–281.

Henry, J.A., Goodworth, M.C., Lima, E., Zaugg, T. & Thielman, E.J. 2022. Cognitive Behavioral Therapy for Tinnitus: Addressing the Controversy of Its Clinical Delivery by Audiologists. *Ear and Hearing* 43(2): 283–289.

Hoare, D.J., Broomhead, E., Stockdale, D. & Kennedy, V. 2015. Equity and person-centeredness in provision of tinnitus services in UK National Health Service audiology departments. *European Journal for Person Centered Healthcare* 3(3): 318.

Hoare, D.J., Kowalkowski, V.L., Kang, S. & Hall, D.A. 2011. Systematic review and meta-analyses of randomized controlled trials examining tinnitus management. *Laryngoscope* 121(7): 1555–1564.

Husain, W.S.W., Zakaria, M.N., Othman, N.A.N., Othman, A., Aw, C.L. & Mohamad, W.N.W. 2020. Management of Subjective Tinnitus by Clinical Professionals in Malaysia: a Cross-Sectional Survey Study. *Hearing Impairment and Disability: Identification, Assessment, and Intervention* 18(2): 183–204.

Jarach, C.M., Lugo, A., Scala, M., Van Den Brandt, P.A., Cederroth, C.R., Odone, A., Garavello, W., Schlee, W., Langguth, B. & Gallus, S. 2022. Global Prevalence and Incidence of Tinnitus: A Systematic Review and Meta-analysis. *JAMA Neurology* 79(9): 888–900.

Julayanont, P., Phillips, N., Chertkow, H. & Nasreddine, Z.S. 2013. Montreal cognitive assessment (MoCA): Concept and clinical review. *Cognitive Screening Instruments: A Practical Approach* (10): 111–151.

Keyworth, C., O'Connor, R., Quinlivan, L. & Armitage, C.J. 2021. Acceptability of a Brief Web-Based Theory-Based Intervention to Prevent and Reduce Self-harm: Mixed Methods Evaluation. *Journal of Medical Internet Research* 23(9): 1–15.

Kooi Cheah, W. 2014. Validation of Malay Version of Montreal Cognitive Assessment in Patients with Cognitive Impairment. *Clinical Medicine Research* 3(3): 56.

Layard, R. 2017. The economics of mental health: With modern psychological therapy, mentally ill people can become more productive and more satisfied with life. *IZA World of Labor* (January): 1–10.

Liu, J., Butow, P. & Beith, J. 2019. Systematic review of interventions by non-mental health specialists for managing fear of cancer recurrence in adult cancer survivors. *Supportive Care in Cancer* 27(11): 4055–4067.

Lovibond, P.F. & Lovibond, S.H. 1995. The structure of negative emotional states: Comparison of the Depression Anxiety Stress Scales (DASS) with the Beck Depression and Anxiety Inventories. *Behaviour Research and Therapy* 33(3): 335–343.

Ma, Y., Hall, D.L., Ngo, L.H., Liu, Q., Bain, P.A. & Yeh, G.Y. 2021, February 1. Efficacy of cognitive behavioral therapy for insomnia in breast cancer: A meta-analysis. *Sleep Medicine Reviews*. W.B. Saunders Ltd.

Mcevoy, P., Landwehr, E., Pearcy, C. & Campbell, B. 2020. A Clinician's Guide to Low Intensity Psychological Interventions (LIPIs) for Anxiety and Depression by WA Primary Health Alliance. *Clinical Interventions* (April).

McKenna, L., Handscomb, L., Hoare, D.J. & Hall, D.A. 2014. A scientific cognitive-behavioral model of tinnitus: Novel conceptualizations of tinnitus distress. *Frontiers in Neurology* 5(OCT).

Meikle, M.B., Henry, J.A., Griest, S.E., Stewart, B.J., Abrams, H.B., McArdle, R., Myers, P.J., Newman, C.W., Sandridge, S., Turk, D.C., Folmer, R.L., Frederick, E.J., House, J.W., Jacobson, G.P., Kinney, S.E., Martin, W.H., Nagler, S.M., Reich, G.E., Searchfield, G., Sweetow, R. & Vernon, J.A. 2012. The tinnitus functional index: Development of a new clinical measure for chronic, intrusive tinnitus. *Ear and Hearing* 33(2): 153–176.

Morrell, C.J., Slade, P., Warner, R., Paley, G., Dixon, S., Walters, S.J., Brugha, T., Barkham, M., Parry, G.J. & Nicholl, J. 2009. Clinical effectiveness of health visitor training in psychologically informed approaches for depression in postnatal women: Pragmatic cluster randomised trial in primary care. *BMJ (Online)* 338(7689): 276–279.

NICE. 2020. Tinnitus: assessment and management NICE guideline (March 2020).

Nolan, D.R., Gupta, R., Huber, C.G. & Schneeberger, A.R. 2020. An Effective Treatment for Tinnitus and Hyperacusis Based on Cognitive Behavioral Therapy in an Inpatient

Setting: A 10-Year Retrospective Outcome Analysis. *Frontiers in Psychiatry* 11(February): 1–8.

Norena, A.J. 2023. The Analogy between Tinnitus and Chronic Pain: A Phenomenological Approach. *Brain Sciences* 13(8).

Oosterloo, B.C., De Feijter, M., Croll, P.H., Baatenburg De Jong, R.J., Luik, A.I. & Goedegebure, A. 2021. Cross-sectional and Longitudinal Associations between Tinnitus and Mental Health in a Population-Based Sample of Middle-aged and Elderly Persons. *JAMA Otolaryngology - Head and Neck Surgery* 147(8): 708–716.

Pinto, P.C.L., Marcelos, C.M., Mezzasalma, M.A., Osterne, F.J.V., de Melo Tavares de Lima, M.A. & Nardi, A.E. 2014. Tinnitus and its association with psychiatric disorders: systematic review. *The Journal of Laryngology and Otology* 128(8): 660–664.

Prosedur Operasi Standard Perkhidmatan Audiologi Dewasa. 2023. Bahagian Sains Kesihatan Bersekutu, Kementerian Kesihatan Malaysia.

Ramli, M., Rosnani, S. & Fasrul, A.A. 2012. Psychometric Profile of Malaysian version of the Depressive, Anxiety and Stress Scale 42-item (DASS-42). *MJP Online Early* 1(1): 7.

Salkovskis, P.M. 1996. The cognitive approach to anxiety: Threat beliefs, safety-seeking behavior, and the special case of health anxiety and obsessions. In *Frontiers of Cognitive Therapy*.

Sekhon, J.K., Douglas, J. & Rose, M.L. 2015. Current Australian speech-language pathology practice in addressing psychological well-being in people with aphasia after stroke. *International Journal of Speech-Language Pathology* 17(3): 252–262.

Sperber, A.D. 2004. Translation and Validation of Study Instruments for Cross-Cultural Research. *Gastroenterology* 126(1): 124–128.

Sweetow, R.W. 1986. Cognitive aspects of tinnitus patient management. *Ear and Hearing* 7(6): 390–396.

Taylor, J.A., Thompson, D.M., Hall, D.A., Walker, D.M., McMurran, M., Casey, A., Featherstone, D., MacDonald, C., Stockdale, D. & Hoare, D.J. 2020. The TinMan study: feasibility trial of a psychologically informed, audiologist-delivered, manualised intervention for tinnitus. *International Journal of Audiology* 0(0): 905–914.

Tunkel, D.E., Bauer, C.A., Sun, G.H., Rosenfeld, R.M., Chandrasekhar, S.S., Cunningham, E.R., Archer, S.M., Blakley, B.W., Carter, J.M., Granieri, E.C., Henry, J.A., Hollingsworth, D., Khan, F.A., Mitchell, S., Monfared, A., Newman, C.W., Omole, F.S., Phillips, C.D., Robinson, S.K., Taw, M.B., Tyler, R.S., Waguespack, R. & Whamond, E.J. 2014. Clinical practice guideline: Tinnitus. *Otolaryngology - Head and Neck Surgery (United States)* 151(2): S1–S40.

Wan Husain, W.S., Zakaria, M.N., Othman, N.A.N., Othman, A., Lih, A.C. & Zainun, Z. 2015. The effectiveness of psychological interventions among tinnitus sufferers: A review. *Medical Journal of Malaysia* 70(3): 188–197.

Wu, V., Cooke, B., Eitutis, S., Simpson, M.T.W. & Beyea, J.A. 2018. Approach to tinnitus management. *Canadian Family Physician* 64(7): 491–495.

7.0 APPENDICES

A psychologically informed guidance manual to support audiologist management of tinnitus

This manual was created as part of a National Institute for Health Research for Patient Benefit funded study (Grant Reference Number PB-PG-0613-31106). The study was approved by the North West – Preston NHS Research Ethics Committee (reference: 16/NW/0047) and Nottingham University Hospitals NHS Trust (Sponsor). The views expressed in the manual are those of the authors and not necessarily those of the NIHR, the NHS or the Department of Health.

The manual resulted from a collaborative effort between patients and patient representatives, audiologist, hearing therapist, cognitive behaviour therapist, and research professionals. The intended purpose of the manual is to provide audiologists with a reference and resources (toolkit) to work with people who have tinnitus in a psychologically informed way to identify individual needs, set joint goals, reach shared and informed decisions, and promote patient self-management. The content is designed to be delivered using a range of counselling skills.

We acknowledge the use of material from the British Tinnitus Association (www.tinnitus.org.uk), Action on Hearing Loss (www.actiononhearingloss.org.uk), NHS Choices (www.nhs.uk), Common Language for Psychotherapy Procedures (www.commonlanguagepsychotherapy.org), Psychology Tools (http://psychology.tools), Get Self Help (www.getselfhelp.co.uk) and the Tinnitus Clinic (www.thetinnitusclinic.co.uk)

© University of Nottingham 2016

Derek Hoare, John Taylor, Dean Thompson, Mary McMurran, Carol MacDonald, Amanda Casey, Debbie Featherstone, Deborah Hall, Dawn-Marie Walker, David Stockdale, Debra Williams, & Hannah Heath (2016) A psychologically informed guidance manual to support audiologist management of tinnitus

6. PATIENT MANAGEMENT/SELF-MANAGEMENT

6.4 CHANGING UNHELPFUL (NEGATIVE) THOUGHTS AND BELIEFS

Aims:

- To reduce the number of unhelpful negative thoughts and beliefs

Rationale:
Tinnitus provokes distress when a person holds overly negative thoughts about it. These negative thoughts provoke physiological arousal and emotional distress that increases attention directed towards tinnitus, making it appear even more of a problem. Inaccurate negative beliefs about the self or tinnitus may also fuel negative thoughts. Such beliefs are based on others' experience of tinnitus or derive from general beliefs about health, the self, or the world. As these conscious cognitive processes have a central role in generating and maintaining tinnitus distress, they are key targets for therapeutic change.

Catastrophising is one style of negative thinking. Catastrophic misinterpretations are a central factor in the maintenance of tinnitus distress and are associated with higher subjective tinnitus loudness. Patients who catastrophically misinterpret their tinnitus are more fearful of, and more vigilant towards, their tinnitus, and experience lower quality of life.

By working through the Thought Record, the patient may be able to develop more helpful things to say about their tinnitus – for example, reminding them that it is not dangerous and it is still possible to enjoy life with tinnitus. Changing the messages they tell themselves about tinnitus can help to reduce its impact. They can step back from negative thoughts more easily, let them go, and allow more helpful thoughts to encourage and guide them towards more helpful actions and feelings. This encourages moving beyond a narrow and unhelpful focus on tinnitus and tinnitus-related activity, and widens the sense of what they can now. With practice, letting go of unhelpful thoughts can become a more natural part of daily life.

Learning Outcomes:
The patient will be able to:

- Describe how unhelpful negative thinking can maintain and worsen tinnitus
- Identify unhelpful negative beliefs and thinking styles that can worsen tinnitus
- List evidence for and against unhelpful negative thoughts
- Identify alternative, realistic, balanced thoughts

Resources:
T17 - Negative Thinking Example Formulation
T18 - Unhelpful Thinking Styles sheet
T19 & T20 - Thought Record: Managing Unhelpful Thoughts, Becoming Less Bothered by Tinnitus (blank & example copies)
T5 - Cognitive Model of Tinnitus psychology tool
(Available in Toolkit)

Negative Thinking Example Formulation

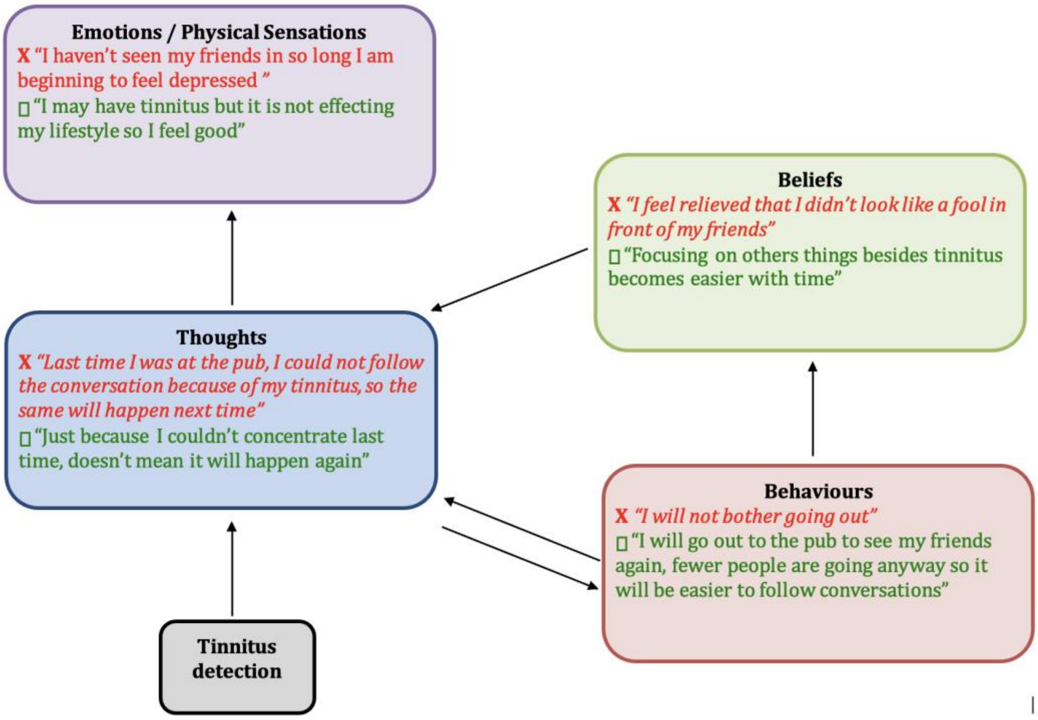

Table 1. Comparability/Interpretability Rating Sheet[15]

Please circle the response which most closely represents how you would rate the following pairs of items in terms of:
(A) Comparability of language (how comparable is the formal wording?) and
(B) Similarity of interpretation (would the paired items be interpreted similarly, even if the wording is different?).
Please circle only one response for (A) and one response for (B) for each pair of items.

		(A) COMPARABILITY OF LANGUAGE						
		EXTREMELY COMPARABLE			MODERATELY COMPARABLE			NOT AT ALL COMPARABLE
		1	2	3	4	5	6	7
Original English version	Back-translated English version	*(B) SIMILARITY OF INTERPRETATION*						
		EXTREMELY SIMILAR			MODERATELY SIMILAR			NOT AT ALL SIMILAR
		1	2	3	4	5	6	7

Adapted from Sperber AD et al.[15]

NAME :
Education : Date of birth :
Sex : DATE :

MONTREAL COGNITIVE ASSESSMENT (MOCA)

Version 7.1 Original Version

VISUOSPATIAL / EXECUTIVE

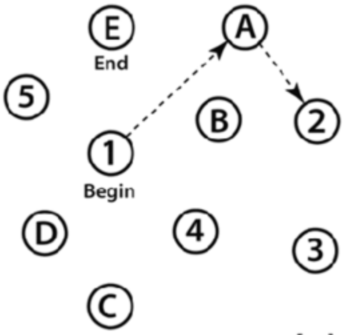

[ ]

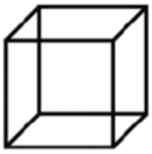

Copy cube

[ ]

Draw CLOCK (Ten past eleven)
(3 points)

[ ] Contour [ ] Numbers [ ] Hands

POINTS: __/5

NAMING

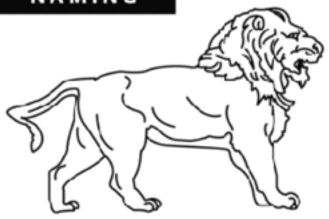
[ ]

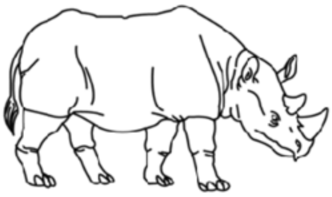
[ ]

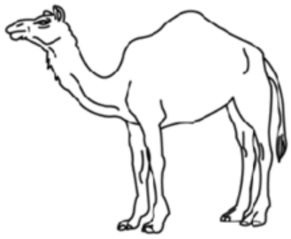
[ ]

__/3

MEMORY

Read list of words, subject must repeat them. Do 2 trials, even if 1st trial is successful. Do a recall after 5 minutes.

	FACE	VELVET	CHURCH	DAISY	RED
1st trial					
2nd trial					

No points

ATTENTION

Read list of digits (1 digit/ sec.). Subject has to repeat them in the forward order [ ] 2 1 8 5 4
Subject has to repeat them in the backward order [ ] 7 4 2 __/2

Read list of letters. The subject must tap with his hand at each letter A. No points if ≥ 2 errors
[ ] F B A C M N A A J K L B A F A K D E A A A J A M O F A A B __/1

Serial 7 subtraction starting at 100 [ ] 93 [ ] 86 [ ] 79 [ ] 72 [ ] 65
4 or 5 correct subtractions: **3 pts**, 2 or 3 correct: **2 pts**, 1 correct: **1 pt**, 0 correct: **0 pt** __/3

LANGUAGE

Repeat : I only know that John is the one to help today. [ ]
The cat always hid under the couch when dogs were in the room. [ ] __/2

Fluency / Name maximum number of words in one minute that begin with the letter F [ ] _____ (N ≥ 11 words) __/1

ABSTRACTION

Similarity between e.g. banana - orange = fruit [ ] train – bicycle [ ] watch - ruler __/2

DELAYED RECALL

Has to recall words	FACE	VELVET	CHURCH	DAISY	RED	Points for UNCUED recall only
WITH NO CUE	[ ]	[ ]	[ ]	[ ]	[ ]	__/5
**Optional** Category cue						
Multiple choice cue						

ORIENTATION

[ ] Date [ ] Month [ ] Year [ ] Day [ ] Place [ ] City __/6

© Z.Nasreddine MD **www.mocatest.org** Normal ≥26 / 30

TOTAL __/30
Add 1 point if ≤ 12 yr edu

Administered by: ________________________

Montreal Cognitive Assessment
– Bahasa Malaysia version

NAMA:
Pendidikan:
Jantina:
Tarikh lahir:
TARIKH:

PENGHAYATAN RUANG/ EKSEKUTIF | **MATA**

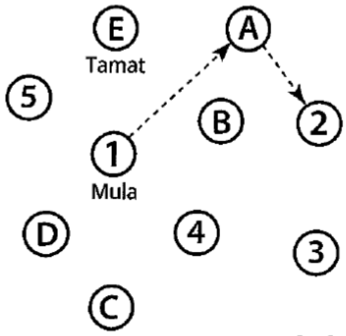

[ ]

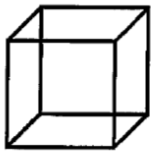

Salin kiub [ ]

Lukis JAM (pukul sebelas sepuluh minit) (3 mata)

[ ] Kontur [ ] Nombor-nombor [ ] Jarum-jarum — ___/5

PENAMAAN

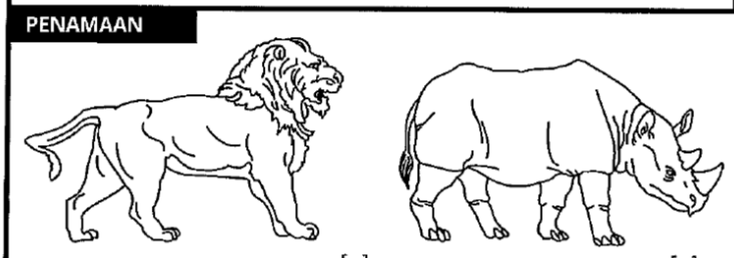

[ ] [ ] [ ] — ___/3

INGATAN Bacakan senarai perkataan, orang yang dinilai mestilah mengulanginya. Lakukan 2 percubaan. Lakukan ingatan kembali selepas 5 minit.

	MUKA	BALDU	GEREJA	BUNGA DAISI	MERAH
Percubaan pertama					
Percubaan ke-2					

Tiada mata

PENUMPUAN Bacakan senarai digit (1 digit/saat)

Orang yang dinilai hendaklah mengulanginya mengikut urutan ke depan [ ] 2 1 8 5 4
Orang yang dinilai hendaklah mengulanginya mengikut urutan ke belakang [ ] 7 4 2 — ___/2

Bacakan senarai huruf. Orang yang dinilai mestilah mengetuk dengan tangannya pada setiap huruf A. Tiada mata jika ≥ 2 kesilapan. [ ] F B A C M N A A J K L B A F A K D E A A A J A M O F A A B — ___/1

Menolak 7 secara bersiri bermula dengan 100 [ ] 93 [ ] 86 [ ] 79 [ ] 72 [ ] 65
4 atau 5 ditolak dengan betul: **3 mata**, 2 atau 3 betul: **2 mata**, 1 betul: **1 mata**, 0 betul: **0 mata** — ___/3

BAHASA Ulang: Saya cuma tahu bahawa John ialah orang yang ditolong. [ ]
Kucing selalu menyorok di bawah kerusi bila anjing berada dalam bilik. [ ] — ___/2

Kelancaran / Namakan bilangan maksimum perkataan dalam satu minit yang bermula dengan huruf A [ ] _____ (N ≥ 11 perkataan) — ___/1

PENGABSTRAKAN Persamaan antara, misalnya, pisang - oren = buah [ ] **kereta api - basikal** [ ] **jam tangan - pembaris** — ___/2

**INGATAN KEMBALI YANG TERTUNDA**	Hendaklah mengingat kembali perkataan TANPA PEMBAYANG	MUKA [ ]	BALDU [ ]	GEREJA [ ]	BUNGA DAISI [ ]	MERAH [ ]	Mata bagi ingatan kembali TANPA PEMBAYANG sahaja	___/5
**Tidak diwajibkan**	Pembayang kategori							
	Pembayang pelbagai pilihan							

ORIENTASI [ ] Tarikh [ ] Bulan [ ] Tahun [ ] Hari [ ] Tempat [ ] Bandaraya — ___/6

© Z.Nasreddine MD www.mocatest.org Normal ≥ 26 / 30 JUMLAH ___/30

Tambah 1 mata jika pendidikan ≤ 12 tahun

Dijalankan oleh: ______________________

Validated by R. Sahathevan et al. Int Psychoger 2014

TINNITUS FUNCTIONAL INDEX

Today's Date ______________ (Month / Day / Year)   Your Name ______________________ (Please Print)

Please read each question below carefully. To answer a question, select *ONE* of the numbers that is listed for that question, and draw a *CIRCLE* around it like this: (10%) or (1).

I	**Over the PAST WEEK...**

1. What percentage of your time awake were you consciously **AWARE OF** your tinnitus?

Never aware ► 0% 10% 20% 30% 40% 50% 60% 70% 80% 90% 100% ◄ *Always aware*

2. How **STRONG** or **LOUD** was your tinnitus?

Not at all strong or loud ► 0 1 2 3 4 5 6 7 8 9 10 ◄ *Extremely strong or loud*

3. What percentage of your time awake were you **ANNOYED** by your tinnitus?

None of the time ► 0% 10% 20% 30% 40% 50% 60% 70% 80% 90% 100% ◄ *All of the time*

SC	**Over the PAST WEEK...**

4. Did you feel **IN CONTROL** in regard to your tinnitus?

Very much in control ► 0 1 2 3 4 5 6 7 8 9 10 ◄ *Never in control*

5. How easy was it for you to **COPE** with your tinnitus?

Very easy to cope ► 0 1 2 3 4 5 6 7 8 9 10 ◄ *Impossible to cope*

6. How easy was it for you to **IGNORE** your tinnitus?

Very easy to ignore ► 0 1 2 3 4 5 6 7 8 9 10 ◄ *Impossible to ignore*

C	**Over the PAST WEEK...**

7. Your ability to **CONCENTRATE**?

Did not interfere ► 0 1 2 3 4 5 6 7 8 9 10 ◄ *Completely interfered*

8. Your ability to **THINK CLEARLY?**

Did not interfere ► 0 1 2 3 4 5 6 7 8 9 10 ◄ *Completely interfered*

9. Your ability to **FOCUS ATTENTION** on other things besides your tinnitus?

Did not interfere ► 0 1 2 3 4 5 6 7 8 9 10 ◄ *Completely interfered*

SL	**Over the PAST WEEK...**

10. How often did your tinnitus make it difficult to **FALL ASLEEP or STAY ASLEEP**?

Never had difficulty ► 0 1 2 3 4 5 6 7 8 9 10 ◄ *Always had difficulty*

11. How often did your tinnitus cause you difficulty in getting **AS MUCH SLEEP** as you needed?

Never had difficulty ► 0 1 2 3 4 5 6 7 8 9 10 ◄ *Always had difficulty*

12. How much of the time did your tinnitus keep you from **SLEEPING** as **DEEPLY** or as **PEACEFULLY** as you would have liked?

None of the time ► 0 1 2 3 4 5 6 7 8 9 10 ◄ *All of the time*

Copyright Oregon Health & Science University 2008 08.15.08

Please read each question below carefully. To answer a question, select *ONE* of the numbers that is listed for that question, and draw a *CIRCLE* around it like this: (10%) or (1).

**A** Over the PAST WEEK, how much has your tinnitus interfered with...	*Did not interfere*										*Completely interfered*
13. Your ability to **HEAR CLEARLY**?	0	1	2	3	4	5	6	7	8	9	10
14. Your ability to **UNDERSTAND PEOPLE** who are talking?	0	1	2	3	4	5	6	7	8	9	10
15. Your ability to **FOLLOW CONVERSATIONS** in a group or at meetings?	0	1	2	3	4	5	6	7	8	9	10

**R** Over the PAST WEEK, how much has your tinnitus interfered with...	*Did not interfere*										*Completely interfered*
16. Your **QUIET RESTING ACTIVITIES**?	0	1	2	3	4	5	6	7	8	9	10
17. Your ability to **RELAX**?	0	1	2	3	4	5	6	7	8	9	10
18. Your ability to enjoy "**PEACE AND QUIET**"?	0	1	2	3	4	5	6	7	8	9	10

**Q** Over the PAST WEEK, how much has your tinnitus interfered with...	*Did not interfere*										*Completely interfered*
19. Your enjoyment of **SOCIAL ACTIVITIES**?	0	1	2	3	4	5	6	7	8	9	10
20. Your **ENJOYMENT OF LIFE**?	0	1	2	3	4	5	6	7	8	9	10
21. Your **RELATIONSHIPS** with family, friends and other people?	0	1	2	3	4	5	6	7	8	9	10

22. How often did your tinnitus cause you to have difficulty performing your **WORK OR OTHER TASKS**, such as home maintenance, school work, or caring for children or others?

Never had difficulty ► 0 1 2 3 4 5 6 7 8 9 10 ◄ *Always had difficulty*

E **Over the PAST WEEK...**

23. How **ANXIOUS** or **WORRIED** has your tinnitus made you feel?

Not at all anxious or worried ► 0 1 2 3 4 5 6 7 8 9 10 ◄ *Extremely anxious or worried*

24. How **BOTHERED** or **UPSET** have you been because of your tinnitus?

Not at all bothered or upset ► 0 1 2 3 4 5 6 7 8 9 10 ◄ *Extremely bothered or upset*

25. How **DEPRESSED** were you because of your tinnitus?

Not at all depressed ► 0 1 2 3 4 5 6 7 8 9 10 ◄ *Extremely depressed*

Copyright Oregon Health & Science University

TINNITUS FUNCTIONAL INDEX-BM (TFI-BM)

Tarikh ___________________ Nama ____________________________

Sila baca setiap soalan di bawah dengan teliti. Untuk menjawab soalan, pilih SATU daripada nombor yang tertulis, dan BULATKAN jawapan seperti berikut: 10% or 1

**I**	**Sepanjang SEMINGGU YANG LEPAS**

1. Berapa peratuskah daripada masa jaga anda yang anda **SEDAR** akan tinitus anda?

   Tidak pernah ◆ 0% 10% 20% 30% 40% 50% 60% 70% 80% 90% 100% ♦ *Sentiasa*

2. Berapa **KUAT** bunyi tinitus anda?

   Tidak kuat langsung ◆ 0 1 2 3 4 5 6 7 8 9 10♦ *Sangat kuat*

3. Berapa peratuskah daripada masa jaga anda yang anda berasa terganggu dengan tinitus anda?

   Tidak pernah ◆ 0% 10% 20% 30% 40% 50% 60% 70% 80% 90% 100% ♦ *Setiap masa*

**SC**	**Sepanjang SEMINGGU YANG LEPAS**

4. Adakah anda merasa anda berada **DALAM KAWALAN** ketika anda mendengar tinnitus anda?

   Sangat dalam kawalan ◆ 0 1 2 3 4 5 6 7 8 9 10 ♦ *Sangat tidak dalam kawalan*

5. Berapa mudah bagi anda untuk menghadapi tinitus anda?

   Sangat mudah ◆ 0 1 2 3 4 5 6 7 8 9 10 ♦ *Mustahil*

6. Berapa mudah bagi anda untuk tidak mempedulikan tinitus anda?

   Sangat mudah ◆ 0 1 2 3 4 5 6 7 8 9 10 ♦ *Mustahil*

**C**	**Sepanjang SEMINGGU YANG LEPAS**

7. Kemampuan anda untuk **MEMBERIKAN TUMPUAN**

   Tidak terganggu ◆ 0 1 2 3 4 5 6 7 8 9 10♦ *Terganggu sepenuhnya*

8. Kemampuan anda untuk **BERFIKIR DENGAN JELAS**

   Tidak terganggu ◆ 0 1 2 3 4 5 6 7 8 9 10♦ *Terganggu sepenuhnya*

9. Kemampuan anda untuk **MEMBERIKAN PERHATIAN** kepada perkara lain selain daripada tinnitus anda?

   Tidak terganggu ◆ 0 1 2 3 4 5 6 7 8 9 10♦ *Terganggu sepenuhnya*

**SL**	**Sepanjang SEMINGGU YANG LEPAS**

10. Berapa kerapkah tinnitus anda menyebabkan anda sukar untuk **TIDUR** atau **TIDUR DENGAN NYENYAK**?

    Tidak pernah menghadapi kesukaran ◆ 0 1 2 3 4 5 6 7 8 9 10♦ *Sering menghadapi kesukaran*

11. Berapa kerapkah tinnitus anda menyukarkan anda mendapat **TIDUR** yang mencukupi seperti yang anda perlukan?

    Tidak pernah mengalami kesukaran ◆ 0 1 2 3 4 5 6 7 8 9 10♦ *Sering mengalami kesukaran*

12. Berapa kerapkah tinnitus anda menghalang anda dari **TIDUR** dengan **NYENYAK** dan **TENANG** seperti yang anda mahu?

Tidak pernah ◆ 0 1 2 3 4 5 6 7 8 9 10♦ *Sentiasa*

A	Sepanjang SEMINGGU YANG LEPAS, sebanyak manakah tinnitus anda mengganggu dari segi…

	Tidak terganggu										*Sangat terganggu*
13. Kemampuan anda **MENDENGAR DENGAN JELAS?**	0	1	2	3	4	5	6	7	8	9	10
14. Kemampuan anda **MEMAHAMI ORANG** lain yang bercakap?	0	1	2	3	4	5	6	7	8	9	10
15. Kemampuan anda untuk **MENGIKUTI PERBUALAN** dalam kumpulan atau perjumpaan?	0	1	2	3	4	5	6	7	8	9	10

R	Sepanjang SEMINGGU YANG LEPAS, sebanyak mana tinnitus anda mengganggu dari segi…

	Tidak terganggu										*Sangat terganggu*
16. **AKTIVITI SENYAP SEMASA** anda **BEREHAT**?	0	1	2	3	4	5	6	7	8	9	10
17. Kemampuan anda untuk **BERSANTAI**?	0	1	2	3	4	5	6	7	8	9	10
18. Kemampuan anda untuk menikmati "**SUASANA YANG AMAN DAN TENANG"**?	0	1	2	3	4	5	6	7	8	9	10

Q	Sepanjang SEMINGGU YANG LEPAS, sebanyak mana tinnitus anda mengganggu…

	Tidak terganggu										*Sangat terganggu*
19. Keseronokan **AKTIVITI SOSIAL** anda?	0	1	2	3	4	5	6	7	8	9	10
20. **KESERONOKAN HIDUP** anda?	0	1	2	3	4	5	6	7	8	9	10
21. **HUBUNGAN** anda dengan keluarga, rakan-rakan dan orang lain?	0	1	2	3	4	5	6	7	8	9	10

22. Berapa kerap tinnitus anda menyebabkan anda menghadapi kesukaran untuk **BEKERJA ATAU** melakukan **TUGAS-TUGAS LAIN** seperti mengurus rumah, menjalankan kerja sekolah, menjaga kanak-kanak dan sebagainya?

Tidak pernah menghadapi kesukaran ◆ 0 1 2 3 4 5 6 7 8 9 10 ♦ *Sering menghadapi kesukaran*

E	Sepanjang SEMINGGU YANG LEPAS

23. Sejauh mana tinnitus anda telah menyebabkan anda berasa **GELISAH** atau **RISAU**?

Tidak gelisah / risau langsung ◆ 0 1 2 3 4 5 6 7 8 9 10 ♦ *Sangat gelisah / risau*

24. Sejauh manakah anda rasa **TERGANGGU** dan **MARAH** disebabkan tinnitus anda?

Tidak terganggu / marah langsung ◆ 0 1 2 3 4 5 6 7 8 9 10 ♦ *Sangat terganggu / marah*

25. Sejauh manakah anda **TERTEKAN** disebabkan oleh tinnitus anda?

Tidak tertekan langsung ◆ 0 1 2 3 4 5 6 7 8 9 10 ♦ *Sangat tertekan*

DASS

Name: *Date:*

Please read each statement and circle a number 0, 1, 2 or 3 which indicates how much the statement applied to you *over the past week*. There are no right or wrong answers. Do not spend too much time on any statement.

The rating scale is as follows:

0 Did not apply to me at all
1 Applied to me to some degree, or some of the time
2 Applied to me to a considerable degree, or a good part of time
3 Applied to me very much, or most of the time

1	I found myself getting upset by quite trivial things	0	1	2	3
2	I was aware of dryness of my mouth	0	1	2	3
3	I couldn't seem to experience any positive feeling at all	0	1	2	3
4	I experienced breathing difficulty (eg, excessively rapid breathing, breathlessness in the absence of physical exertion)	0	1	2	3
5	I just couldn't seem to get going	0	1	2	3
6	I tended to over-react to situations	0	1	2	3
7	I had a feeling of shakiness (eg, legs going to give way)	0	1	2	3
8	I found it difficult to relax	0	1	2	3
9	I found myself in situations that made me so anxious I was most relieved when they ended	0	1	2	3
10	I felt that I had nothing to look forward to	0	1	2	3
11	I found myself getting upset rather easily	0	1	2	3
12	I felt that I was using a lot of nervous energy	0	1	2	3
13	I felt sad and depressed	0	1	2	3
14	I found myself getting impatient when I was delayed in any way (eg, elevators, traffic lights, being kept waiting)	0	1	2	3
15	I had a feeling of faintness	0	1	2	3
16	I felt that I had lost interest in just about everything	0	1	2	3
17	I felt I wasn't worth much as a person	0	1	2	3
18	I felt that I was rather touchy	0	1	2	3
19	I perspired noticeably (eg, hands sweaty) in the absence of high temperatures or physical exertion	0	1	2	3
20	I felt scared without any good reason	0	1	2	3
21	I felt that life wasn't worthwhile	0	1	2	3

Please turn the page ☞

Reminder of rating scale:

0 Did not apply to me at all
1 Applied to me to some degree, or some of the time
2 Applied to me to a considerable degree, or a good part of time
3 Applied to me very much, or most of the time

22	I found it hard to wind down	0	1	2	3
23	I had difficulty in swallowing	0	1	2	3
24	I couldn't seem to get any enjoyment out of the things I did	0	1	2	3
25	I was aware of the action of my heart in the absence of physical exertion (eg, sense of heart rate increase, heart missing a beat)	0	1	2	3
26	I felt down-hearted and blue	0	1	2	3
27	I found that I was very irritable	0	1	2	3
28	I felt I was close to panic	0	1	2	3
29	I found it hard to calm down after something upset me	0	1	2	3
30	I feared that I would be "thrown" by some trivial but unfamiliar task	0	1	2	3
31	I was unable to become enthusiastic about anything	0	1	2	3
32	I found it difficult to tolerate interruptions to what I was doing	0	1	2	3
33	I was in a state of nervous tension	0	1	2	3
34	I felt I was pretty worthless	0	1	2	3
35	I was intolerant of anything that kept me from getting on with what I was doing	0	1	2	3
36	I felt terrified	0	1	2	3
37	I could see nothing in the future to be hopeful about	0	1	2	3
38	I felt that life was meaningless	0	1	2	3
39	I found myself getting agitated	0	1	2	3
40	I was worried about situations in which I might panic and make a fool of myself	0	1	2	3
41	I experienced trembling (eg, in the hands)	0	1	2	3
42	I found it difficult to work up the initiative to do things	0	1	2	3

DASS

Nama: *Tarikh:*

Sila baca setiap kenyataan di bawah dan bulatkan pada nombor 0,1,2 atau 3 bagi menggambarkan keadaan anda sepanjang minggu yang lalu. Tiada jawapan yang betul atau salah. Jangan mengambil masa yang terlalu lama untuk menjawab mana-mana kenyataan.

Skala pemarkahan adalah seperti berikut:

0 **Tidak langsung** menggambarkan keadaan saya
1 **Sedikit atau jarang-jarang** menggambarkan keadaan saya.
2 **Banyak atau kerapkali** menggambarkan keadaan saya.
3 **Sangat banyak atau sangat kerap** menggambarkan keadaan saya

1	Saya dapati diri saya menjadi kesal/marah disebabkan perkara-perkara yang kecil.	0	1	2	3
2	Saya sedar mulut saya terasa kering	0	1	2	3
3	Saya tidak dapat mengalami perasaan positif sama sekali	0	1	2	3
4	Saya mengalami kesukaran bernafas (contohnya pernafasan yang laju, tercungap-cungap walaupun tidak melakukan senaman fizikal)	0	1	2	3
5	Saya rasa diri saya tidak bergerak ke mana-mana	0	1	2	3
6	Saya cenderung untuk bertindak keterlaluan dalam sesuatu keadaan	0	1	2	3
7	Saya mempunyai perasaan gementar (seperti kaki menjadi lemah)	0	1	2	3
8	Saya rasa sukar untuk relaks	0	1	2	3
9	Saya dapati diri saya di dalam keadaan yang menjadikan saya amat risau dan menjadi tenang semula selepas ianya berakhir	0	1	2	3
10	Saya rasa saya tidak mempunyai apa-apa untuk diharapkan	0	1	2	3
11	Saya dapati diri saya mudah merasa kesal	0	1	2	3
12	Saya rasa saya menggunakan banyak tenaga dalam keadaan cemas	0	1	2	3
13	Saya rasa sedih dan murung	0	1	2	3
14	Saya dapati diri saya hilang kesabaran sekiranya saya dilambatkan oleh sesuatu (seperti lif, lampu trafik, terpaksa lama menunggu)	0	1	2	3
15	Saya rasa macam nak pengsan	0	1	2	3
16	Saya rasa saya hilang minat dalam segala hal	0	1	2	3
17	Saya tidak begitu berharga sebagai seorang individu	0	1	2	3
18	Saya rasa yang saya mudah tersentuh	0	1	2	3
19	Saya banyak berpeluh (contohnya pada tangan) walaupun bukan pada suhu tinggi atau tiada pergerakan fizikal.	0	1	2	3
20	Saya berasa takut tanpa sebab yang munasabah	0	1	2	3
21	Saya rasa hidup ini sudah tidak bermakna lagi	0	1	2	3

Translation by Dr Ramli Musa

Sila lihat muka surat sebelah ☞

Ingatan skala permarkahan:

0 **Tidak langsung** menggambarkan keadaan saya
1 **Sedikit atau jarang-jarang** menggambarkan keadaan saya.
2 **Banyak atau kerapkali** menggambarkan keadaan saya.
3 **Sangat banyak atau sangat kerap** menggambarkan keadaan saya

22	Saya dapati diri saya sukar ditenteramkan	0	1	2	3
23	Saya rasa sukar menelan	0	1	2	3
24	Saya tidak dapat merasakan keseronokan dalam apa yang saya lakukan	0	1	2	3
25	Saya sedar tindakbalas jantung saya walaupun tidak melakukan aktiviti fizikal (contohnya kadar denyutan jantung bertambah, atau denyutan jantung berkurangan)	0	1	2	3
26	Saya rasa duka dan tidak keruan	0	1	2	3
27	Saya dapati diri saya mudah marah	0	1	2	3
28	Saya rasa hampir-hampir menjadi panik/cemas	0	1	2	3
29	Saya dapati sukar untuk bertenang setelah sesuatu membuatkan saya kesal	0	1	2	3
30	Saya risau saya akan 'dihambat' oleh tugas yang remeh dan tidak biasa dilakukan	0	1	2	3
31	Saya tidak bersemangat dengan apa jua yang saya lakukan	0	1	2	3
32	Saya sukar bersabar pada gangguan terhadap perkara yang sedang saya lakukan	0	1	2	3
33	Saya di dalam keadaan yang terlalu gementar	0	1	2	3
34	Saya rasa diri saya langsung tidak berharga	0	1	2	3
35	Saya hilang pertimbangan pada perkara yang menghalang saya meneruskan apa yang saya lakukan	0	1	2	3
36	Saya rasa amat takut	0	1	2	3
37	Saya melihat tiada masa depan untuk saya menaruh harapan	0	1	2	3
38	Saya rasa hidup ini tidak bermakna	0	1	2	3
39	Saya dapati diri saya semakin gelisah	0	1	2	3
40	Saya bimbang keadaan di mana saya mungkin menjadi panik dan melakukan perkara yang membodohkan diri sendiri	0	1	2	3
41	Saya rasa menggeletar (contohnya pada tangan)	0	1	2	3
42	Saya sukar untuk mendapatkan semangat bagi melakukan sesuatu perkara	0	1	2	3

Table 1 Generic form of TFA acceptability questionnaire

<table>
<tr><td>TFA Construct</td><td>Questionnaire item</td></tr>
<tr><td>Affective attitude
How an individual feels about the intervention</td><td>Affective attitude:
Did you like or dislike [intervention]?
<table><tr><td>Strongly dislike</td><td>Dislike</td><td>No opinion</td><td>Like</td><td>Strongly like</td></tr><tr><td>1</td><td>2</td><td>3</td><td>4</td><td>5</td></tr></table>
OR
How comfortable did you feel [behaviour e.g. to engage with] [intervention]?
<table><tr><td>Very uncomfortable</td><td>Uncomfortable</td><td>No opinion</td><td>Comfortable</td><td>Very comfortable</td></tr><tr><td>1</td><td>2</td><td>3</td><td>4</td><td>5</td></tr></table></td></tr>
<tr><td>Burden
The amount of effort required to participate in the intervention</td><td>How much effort did it take [behaviour e.g. to engage with] [intervention]?
<table><tr><td>No effort at all</td><td>A little effort</td><td>No opinion</td><td>A lot of effort</td><td>Huge effort</td></tr><tr><td>1</td><td>2</td><td>3</td><td>4</td><td>5</td></tr></table></td></tr>
<tr><td>Ethicality
The extent to which the intervention has good fit with an individual's value system</td><td>Ethical Consequences:
There are moral or ethical consequences [behaviour e.g. to engage with] [intervention]
<table><tr><td>Strongly disagree</td><td>Disagree</td><td>No opinion</td><td>Agree</td><td>Strongly agree</td></tr><tr><td>1</td><td>2</td><td>3</td><td>4</td><td>5</td></tr></table>
OR
How fair is [Intervention] for [people/ participants/ recipients] with [condition]?
<table><tr><td>Very unfair</td><td>Unfair</td><td>No opinion</td><td>Fair</td><td>Very fair</td></tr><tr><td>1</td><td>2</td><td>3</td><td>4</td><td>5</td></tr></table></td></tr>
<tr><td>Perceived effectiveness
The extent to which the intervention is have achieved its intended purpose</td><td>The [intervention] has improve [behaviour/ condition/ clinical outcome]:
<table><tr><td>Strongly disagree</td><td>Disagree</td><td>No opinion</td><td>Agree</td><td>Strongly agree</td></tr><tr><td>1</td><td>2</td><td>3</td><td>4</td><td>5</td></tr></table></td></tr>
<tr><td>Intervention coherence
The extent to which the participant understands how the intervention works</td><td>It is clear to me how [intervention] will help [manage/ improve] my [behaviour/ condition/clinical outcome]
<table><tr><td>Strongly disagree</td><td>Disagree</td><td>No opinion</td><td>Agree</td><td>Strongly agree</td></tr><tr><td>1</td><td>2</td><td>3</td><td>4</td><td>5</td></tr></table>
*Please tell us more about your views</td></tr>
<tr><td>Self -efficacy
The participants confidence that they can perform behaviour(s) required to participate in the intervention</td><td>Self-efficacy:
How confident did you feel about [behaviour e.g. engaging with] [intervention]?
<table><tr><td>Very unconfident</td><td>Unconfident</td><td>No opinion</td><td>Confident</td><td>Very confident</td></tr><tr><td>1</td><td>2</td><td>3</td><td>4</td><td>5</td></tr></table></td></tr>
<tr><td>Opportunity costs
The benefits, profits or values that were given up to engage in the intervention</td><td>Opportunity Costs:
[Behaviour e.g. engaging in] [intervention] interfered with my other priorities
<table><tr><td>Strongly disagree</td><td>Disagree</td><td>No opinion</td><td>Agree</td><td>Strongly agree</td></tr><tr><td>1</td><td>2</td><td>3</td><td>4</td><td>5</td></tr></table></td></tr>
<tr><td>General Acceptability</td><td>How acceptable was the [intervention] to you?
<table><tr><td>Completely unacceptable</td><td>Unacceptable</td><td>No opinion</td><td>Acceptable</td><td>Completely acceptable</td></tr><tr><td>1</td><td>2</td><td>3</td><td>4</td><td>5</td></tr></table></td></tr>
</table>

Note: [intervention] should be replaced with the name of the healthcare intervention (e.g. Did you like or dislike the [feedback materials]? [Engaging with/ engaging with intervention] should be replaced with the specific behaviour participants are required to complete to engage with the intervention (e.g. how much effort did it to take you to [book your own appointment]? [clinical condition] should be replaced with the name of the clinical condition associated with the intervention (e.g. it is clear to me how [feedback reports] will result in improvements in [blood transfusion practice].

MOH/P/BSKB/14.23(GU)-e

KEMENTERIAN KESIHATAN MALAYSIA

PROSEDUR OPERASI STANDARD

PERKHIDMATAN AUDIOLOGI DEWASA

HELAIAN MAKLUMAT PESERTA

Tajuk Kajian

Keberkesanan Intervensi Psikologi Berkeamatan Rendah yang Disampaikan oleh Audiologis Versi Adaptasi dalam Membantu Mengurangkan Tahap Keterukan dan Kesukaran yang Dihadapi Pesakit Tinitus Kronik di Malaysia.

Pendahuluan:

Anda dijemput untuk menyertai fasa pertama kajian ini, iaitu penilaian manual intervensi yang diterjemah kepada Bahasa Melayu. Sebelum anda mengambil bahagian dalam kajian ini, adalah penting untuk anda mengambil masa untuk membaca dan memahami Helaian Maklumat Peserta ini.

Tujuan Kajian:

Kajian menunjukkan pesakit yang mengalami masalah tinitus, iaitu gejala bunyi berdesing dalam telinga/kepala yang kronik (melebihi 6 bulan) juga menghadapi kesan psikologi seperti kemurungan, kebimbangan dan tekanan. Intervensi psikologi yang disampaikan oleh psikologis telah terbukti berkesan dalam merawat pesakit tinitus kronik. Walau bagaimanapun, pesakit tinitus kebanyakannya dirujuk kepada audiologis disebabkan perkaitannya dengan masalah pendengaran. Oleh yang demikian, kajian ini ingin menilai keberkesanan intervensi psikologi yang disampaikan oleh audiologis bagi mengurangkan tahap keterukan tinitus pesakit.

Bagaimanakah kajian ini dijalankan?

'*A Psychologically Informed Guidance Manual to Support Audiologist Management of Tinnitus*' yang dihasilkan oleh Taylor dan rakan-rakan di NIHR Nottingham Biomedical Research Centre pada tahun 2020 akan diterjemah kepada Bahasa Melayu. Selepas proses penterjemahan, bahan-bahan sokongan dilampirkan bersama manual intervensi, bertujuan membantu penyampaian dan pembelajaran modul pengurusan oleh pesakit ketika mengikuti proses tersebut bersama-sama dengan audiologis, akan dinilai mengikut dua kriteria: kebolehbacaan dan kesesuaian.

Kebolehbacaan akan dinilai menggunakan formula sementara kesesuaian akan dinilai oleh 8 pesakit tinitus kronik. Pesakit diminta untuk membaca keseluruhan bahan sokongan yang telah diterjemah. Selepas itu, pesakit-pesakit akan ditemubual dengan mengemukakan soalan tentang bahan-bahan sokongan mengikut empat kriteria: 1) Tarikan, 2) Pemahaman, 3) Keberkesanan Kendiri, dan 4) Kesesuaian Budaya.

Faedah kajian:

Dengan menyertai kajian percubaan ini, anda akan membantu dalam menyiapkan manual intervensi dengan memastikan kandungannya bersesuaian dengan masyarakat Malaysia. Intervensi yang telah diadaptasi akan digunakan dalam fasa kedua kajian, iaitu dengan menjalankan percubaan kebolehlaksanaan bagi menentukan keberkesanan dan penerimaannya dalam kalangan pesakit tinnitus kronik, sebelum boleh digunakan secara selamat di Malaysia.

Risiko Kajian

Peserta tidak akan mengalami sebarang risiko jika menyertai kajian ini. Segala maklumat yang diberi adalah sulit.

Adakah anda perlu menyertai kajian ini?

Penyertaan dalam kajian ini adalah secara sukarela. Sekiranya anda bersetuju untuk mengambil bahagian, anda akan diminta untuk menandatangani "Surat Kebenaran". Anda akan diberikan salinan surat kebenaran tersebut dan Borang Maklumat Subjek ini.

Rawatan anda tidak akan terjejas sekiranya anda memutuskan untuk tidak mengambil bahagian dalam kajian ini. Rawatan tinitus anda akan terus diberikan mengikut Prosedur Operasi Standard Perkhidmatan Audiologi Dewasa, Kementerian Kesihatan Malaysia.

Sekiranya anda telah mengambil bahagian, anda masih boleh menarik diri daripada kajian tanpa sebarang denda. Data yang sedia ada mungkin akan digunakan bagi memenuhi manfaat kajian.

Maklumat Sulit:

Maklumat dalam kajian ini akan dibuat dalam bentuk laporan yang akan diterbitkan. Maklumat-maklumat ini hanya boleh diakses oleh para pengkaji dan Jawatankuasa Etika Penyelidikan Universiti Kebangsaan Malaysia. Maklumat ini akan dilaporkan dalam bentuk kolektif dan tidak akan merujuk kepada individu tertentu. Dengan ini maklumat anda adalah sulit dan terpelihara.

Bayaran dan pampasan:

Anda tidak perlu membayar ataupun dibayar untuk menyertai kajian ini. Anda hanya perlu membayar bil rawatan hospital seperti biasa.

Kepada siapakah yang saya boleh bertanya tentang kajian ini?

Sekiranya anda mempunyai sebarang persoalan, anda boleh merujuk kepada Kumpulan Penyelidik. Anda juga boleh menghubungi Jawatankuasa Etika Penyelidikan UKM untuk pengesahan.

Kumpulan Penyelidik:

Nama: Dr Wan Syafira Ishak (Penyelia Utama)
Program Audiologi, Pusat Kajian Penuaan Sihat & Kesejahteraaan (H-CARE), Fakulti Sains Kesihatan.
Nombor Telefon: 03-9289 5005

Nama: Mohamad Azmeer bin Sadali (Pelajar Sarjana)
Program Audiologi, Pusat Kajian Penuaan Sihat & Kesejahteraaan (H-CARE), Fakulti Sains Kesihatan.
Nombor Telefon: 013-755 5684

PARTICIPANT INFORMATION SHEET

Research Title:
Efficacy of the Adapted Version of Audiologist-Delivered Low Intensity Psychological Intervention in Improving Severity and Distress among Chronic Tinnitus Patients in Malaysia.

Introduction:
You are invited to participate in the first phase of the study, which is piloting the culturally adapted intervention manual. Before taking part in this study, it is important that you take time to read and understand the information in this Information Sheet.

Purpose of Study:

Previous studies showed that patients with chronic tinnitus, a condition characterized by perception of ringing sound in the ears/head that persists over 6 months, also experiencing psychological impacts such as depression, anxiety, and stress. Psychological interventions delivered by psychologists, are the leading evidence in treating chronic tinnitus. Nevertheless, tinnitus patients are mostly referred to audiologists as tinnitus is commonly a symptom to hearing problems. Therefore, the aim of this study is to evaluate the efficacy of low intensity psychological intervention delivered by audiologists in reducing tinnitus severity and distress.

What will the study involve?

'A Psychologically Informed Guidance Manual to Support Audiologist Management of Tinnitus' developed by Taylor and colleagues at NIHR Nottingham Biomedical Research Centre in 2020 will be adapted to Malaysian culture. After the adaptation, the accompanying toolkits of resources, aimed to aid the delivery of tinnitus management and enable learning by the patients as they work through the process in partnership with the audiologist, will be evaluated into two criteria: readability and suitability.

Readability will be assessed using formulas while suitability will be assessed by 8 chronic tinnitus patients. The patients will be requested to read the whole adapted toolkits of resources. After that, patients will be interviewed by asking questions about the toolkits of resources that incorporated into four criteria: 1) Attraction, 2) Comprehension, 3) Self-efficacy and 4) Cultural appropriateness.

Benefits:

By participating in this pilot study, you will help in finalizing the intervention manual by ensuring it is culturally appropriate among Malaysian population. The adapted intervention will be utilized in the second phase of the study which is conducting a feasibility trial to determine its efficacy and acceptability among chronic tinnitus patients, before can be safely practiced in Malaysia.

Risks

There is no risk imposed to participants while taking part in this study. All participant information will be kept confidential.

Do you have to take part?

Participation in this study is voluntary. If you agree to take part, then you will be asked to sign the "Informed Consent Form". You will be given a copy of the form and this Information Sheet.

Your treatment is not affected if you decide not to participant in this study. You will undergo standard care adhering to Standard Operational Procedures of Audiological Services for Adults by the Ministry of Health, Malaysia.

Should you decide to participate, you can still withdraw from the study without penalty. In this event, you will not be penalised or lose your rights as a patient. Your data can still be potentially used for the benefits of this study.

Data & Confidentiality:

The data from this study will be made into a report which may be published. Access to the data is only by the research team and the REC UKM. The data will be reported in a collective manner with no reference to an individual. Hence your identity will be kept confidential.

Payment and compensation:

You do not have to pay, nor will you be paid to participate in this study. You do have to pay for the usual hospital charges.

Who can I ask about the study?

If you have any questions, you can direct them to the research team. You can also contact the REC UKM for clarifications.

Name: Dr Wan Syafira binti Ishak (Principal Investigator)
Audiology Program, Centre for Healthy Ageing & Wellness (H-CARE), Faculty of Health Sciences, University Kebangsaan Malaysia.
Phone Number: 03-9289 5005

Name: Mohamad Azmeer bin Sadali (Postgraduate Student)
Audiology Program, Centre for Healthy Ageing & Wellness (H-CARE), Faculty of Health Sciences, University Kebangsaan Malaysia.
Phone Number: 013-755 5684

HELAIAN MAKLUMAT PESERTA

Tajuk Kajian

Keberkesanan Intervensi Psikologi Berkeamatan Rendah yang Disampaikan oleh Audiologis Versi Adaptasi dalam Membantu Mengurangkan Tahap Keterukan Dan Kesukaran yang Dihadapi Pesakit Tinitus Kronik di Malaysia.

Pendahuluan:

Anda dijemput untuk menyertai fasa kedua kajian ini, iaitu percubaan kebolehlaksanaan intervensi psikologi yang telah diadaptasi semasa fasa pertama. Sebelum anda mengambil bahagian dalam kajian ini, adalah penting untuk anda mengambil masa untuk membaca dan memahami Helaian Maklumat Peserta ini.

Tujuan Kajian:

Kajian menunjukkan pesakit yang mengalami masalah tinitus, iaitu gejala bunyi berdesing dalam telinga/kepala yang kronik (melebihi 6 bulan) juga menghadapi kesan psikologi seperti kemurungan, kebimbangan dan tekanan. Intervensi psikologi yang disampaikan oleh psikologis telah terbukti berkesan dalam merawat pesakit tinitus kronik. Walau bagaimanapun, pesakit tinitus kebanyakannya dirujuk kepada audiologis disebabkan perkaitannya dengan masalah pendengaran. Oleh yang demikian, kajian ini ingin menilai keberkesanan intervensi psikologi yang disampaikan oleh audiologis bagi mengurangkan tahap keterukan tinitus pesakit.

Bagaimanakah kajian ini dijalankan?

Anda akan disaring dahulu dengan penilaian kognitif bagi mengenal pasti masalah keupayaan berfikir atau ingatan. Sekiranya tiada masalah kognitif, anda seterusnya akan ditugaskan secara rawak kepada satu dari dua kumpulan: kumpulan intervensi atau kumpulan kawalan menggunakan perisian komputer. Rawak bermaksud setiap peserta mempunyai peluang yang sama rata untuk dtugaskan kepada mana-mana kumpulan.

Kumpulan intervensi akan menerima intervensi psikologi berkeamatan rendah yang disampaikan oleh audiologis, manakala kumpulan kawalan akan menerima rawatan standard. Melalui borang-borang soal selidik yang disediakan, peserta dalam kedua-dua kumpulan akan dipantau tahap keterukan tinitus, kemurungan, kebimbangan dan tekanan pada tiga garis masa: sebelum menerima rawatan, sejurus selepas menerima rawatan dan 3 bulan selepas menerima rawatan.

Di akhir kajian, peserta dalam kumpulan intervensi akan ditemuramah bagi mengetahui sejauh mana penerimaan mereka terhadap intervensi tersebut. Peserta dalam kumpulan kawalan pula akan ditawarkan dengan intervensi yang dikaji sekiranya terbukti berkesan.

Faedah kajian:

Sekiranya intervensi psikologi berkeamatan rendah yang disampaikan oleh audiologis terbukti berkesan, satu garis panduan standard akan dihasilkan bagi kegunaan audiologis-audiologis di seluruh Malaysia untuk membantu pesakit tinitus. Intervensi ini sekiranya dapat dilaksanakan, tidak semua pesakit tinitus perlu dirujuk kepada psikologis/psikiatris seterusnya berpotensi mengurangkan beban

pembiayaan kos rawatan. Walau bagaimanapun, pesakit tinnitus yang mengalami masalah psikologi yang serius masih perlu berjumpa dengan pengamal kesihatan mental.

Risiko Kajian

Peserta tidak akan mengalami sebarang risiko jika menyertai kajian ini. Segala maklumat yang diberi adalah sulit.

Adakah anda perlu menyertai kajian ini?

Penyertaan dalam kajian ini adalah secara sukarela. Sekiranya anda bersetuju untuk mengambil bahagian, anda akan diminta untuk menandatangani "Surat Kebenaran". Anda akan diberikan salinan surat kebenaran tersebut dan Borang Maklumat Subjek ini.

Rawatan anda tidak akan terjejas sekiranya anda memutuskan untuk tidak mengambil bahagian dalam kajian ini. Rawatan tinitus anda akan terus diberikan mengikut Prosedur Operasi Standard Perkhidmatan Audiologi Dewasa, Kementerian Kesihatan Malaysia.

Sekiranya anda telah mengambil bahagian, anda masih boleh menarik diri daripada kajian tanpa sebarang denda. Data yang sedia ada mungkin akan digunakan bagi memenuhi manfaat kajian.

Maklumat Sulit:

Maklumat dalam kajian ini akan dibuat dalam bentuk laporan yang akan diterbitkan. Maklumat-maklumat ini hanya boleh diakses oleh para pengkaji dan Jawatankuasa Etika Penyelidikan Universiti Kebangsaan Malaysia. Maklumat ini akan dilaporkan dalam bentuk kolektif dan tidak akan merujuk kepada individu tertentu. Dengan ini maklumat anda adalah sulit dan terpelihara.

Bayaran dan pampasan:

Anda tidak perlu membayar ataupun dibayar untuk menyertai kajian ini. Anda hanya perlu membayar bil rawatan hospital seperti biasa.

Kepada siapakah yang saya boleh bertanya tentang kajian ini?

Sekiranya anda mempunyai sebarang persoalan, anda boleh merujuk kepada Kumpulan Penyelidik. Anda juga boleh menghubungi Jawatankuasa Etika Penyelidikan UKM untuk pengesahan.

Kumpulan Penyelidik:

Nama: Dr Wan Syafira Ishak (Penyelia Utama)
Program Audiologi, Pusat Kajian Penuaan Sihat & Kesejahteraaan (H-CARE), Fakulti Sains Kesihatan.
Nombor Telefon: 03-9289 5005

Nama: Mohamad Azmeer bin Sadali (Pelajar Sarjana)
Program Audiologi, Pusat Kajian Penuaan Sihat & Kesejahteraaan (H-CARE), Fakulti Sains Kesihatan.
Nombor Telefon: 013-755 5684

PARTICIPANT INFORMATION SHEET

Research Title:
Efficacy of the Adapted Version of Audiologist-Delivered Low Intensity Psychological Intervention in Improving Severity and Distress among Chronic Tinnitus Patients in Malaysia.

Introduction:
You are invited to participate in second phase of the study, which is the feasibility trial of the psychological intervention adapted during the first phase. Before taking part in this study, it is important that you take time to read and understand the information in this Information Sheet.

Purpose of Study:

Previous studies showed that patients with chronic tinnitus, a condition characterized by perception of ringing sound in the ears/head that persists over 6 months, also experiencing psychological impacts such as depression, anxiety, and stress. Psychological interventions delivered by psychologists, are the leading evidence in treating chronic tinnitus. Nevertheless, tinnitus patients are mostly referred to audiologists as tinnitus is commonly a symptom to hearing problems. Therefore, the aim of this study is to evaluate the efficacy of low intensity psychological intervention delivered by audiologists in reducing tinnitus severity.

What will the study involve?

If you decide to participate, you will be first screened for mild cognitive impairment. Having cognitive impairment ruled out, you will then be randomly assigned to one of two groups: the intervention group or the control group using a computer software. Randomization means that each participant has an equal chance of being assigned to either group.

The intervention group will receive audiologist-delivered low intensity psychological intervention, while the control group will receive standard audiological care. Using valid questionnaires, participants in both groups will be monitored their tinnitus severity and the levels of depression, anxiety, and stress at 3 different timelines: before the intervention, at the end of intervention, and 3-month follow-up after the intervention.

At the end of the study, participants in the intervention group will be interviewed to gather insights into how acceptable the intervention is. Meanwhile, participants in the control group will be offered with the intervention if its efficacy is proven.

Benefits:

If audiologist-delivered low intensity psychological intervention is proven efficacious, a standard guideline can be produced for usage among audiologists to better help chronic tinnitus patients in Malaysia. This available intervention, if performed, may reduce unnecessary referrals to psychologists or psychiatrists, hence potentially cost effective to tinnitus patients. However, those with associated severe mental health needs should still require a formal session with mental health specialists.

Risks

There is no risk imposed to participants while taking part in this study. All participant information will be kept confidential.

Do you have to take part?

Participation in this study is voluntary. If you agree to take part, then you will be asked to sign the "Informed Consent Form". You will be given a copy of the form and this Information Sheet.

Your treatment is not affected if you decide not to participant in this study. You will undergo standard care adhering to Standard Operational Procedures of Audiological Services for Adults by the Ministry of Health, Malaysia.

Should you decide to participate, you can still withdraw from the study without penalty. In this event, you will not be penalised or lose your rights as a patient. Your data can still be potentially used for the benefits of this study.

Data & Confidentiality:

The data from this study will be made into a report which may be published. Access to the data is only by the research team and the REC UKM. The data will be reported in a collective manner with no reference to an individual. Hence your identity will be kept confidential.

Payment and compensation:

You do not have to pay, nor will you be paid to participate in this study. You do have to pay for the usual hospital charges.

Who can I ask about the study?

If you have any questions, you can direct them to the research team. You can also contact the REC UKM for clarifications.

Name: Dr Wan Syafira binti Ishak (Principal Investigator)
Audiology Program, Centre for Healthy Ageing & Wellness (H-CARE), Faculty of Health Sciences, University Kebangsaan Malaysia.
Phone Number: 03-9289 5005

Name: Mohamad Azmeer bin Sadali (Postgraduate Student)
Audiology Program, Centre for Healthy Ageing & Wellness (H-CARE), Faculty of Health Sciences, University Kebangsaan Malaysia.
Phone Number: 013-755 5684

BORANG KEIZINAN PESERTA

Tajuk Kajian: Keberkesanan Intervensi Psikologi Berkeamatan Rendah yang Disampaikan oleh Audiologis Versi Adaptasi dalam Membantu Mengurangkan Tahap Keterukan Dan Kesukaran yang Dihadapi Pesakit Tinitus Kronik di Malaysia.

Nama Penyelidik: Dr Wan Syafira binti Ishak, PM Dr Mahadir bin Ahmad, Mohamad Azmeer bin Sadali

Saya, ..., Nombor Kad Pengenalan : ....

- telah membaca maklumat dalam helaian maklumat peserta termasuk maklumat berkaitan risiko dalam kajian ini.
- telah diberikan masa untuk berfikir mengenainya dan semua soalan-soalan saya telah dijawab dengan memuaskan.
- memahami bahawa saya bebas boleh memilih untuk menarik diri daripada kajian ini pada bila-bila masa tanpa sebab dan tanpa apa-apa akibat
- memahami yang nama saya tidak akan disiarkan di dalam penulisan kajian.

Saya secara sukarela bersetuju untuk menjadi sebahagian daripada kajian penyelidikan ini, mematuhi prosedur kajian, dan untuk menyediakan maklumat yang diperlukan oleh ahli jawatankuasa penyelidikan, seperti yang diminta.

………………………………. **…………………..**
(Tandatangan) **(Tarikh)**

………………………………<br>Nama Saksi (sekiranya ada)	……………………………….<br>Nama Penyelidik
………………………………<br>(Tandatangan)	……………………………….<br>(Tandatangan)
………………………………<br>(No. IC)	……………………………….<br>No. IC)
…………………………..…<br>(Tarikh)	……………………………….<br>(Tarikh)

PARTICIPANT INFORMED CONSENT FORM

Research Title: Efficacy of the Adapted Version of Audiologist-Delivered Low Intensity Psychological Intervention in Improving Severity and Distress among Chronic Tinnitus Patients in Malaysia.

Researcher's Name: Dr Wan Syafira Ishak, PM Dr Mahadir Ahmad, Mohamad Azmeer Sadali

I, ……………………………………., IC No : ……………………......

- have read the information in the Participant Information Sheet **including information regarding the risk in this study.**
- have been given time to think about it and all of my questions have been answered to my satisfaction.
- understand that I may freely choose to withdraw from this study at anytime without reason and without repercussion
- understand that my anonymity will be ensured in the write-up.

I voluntarily agree to be part of this research study, to follow the study procedures, and to provide necessary information to the research members, as requested.

……………………………… ………………….
(Signature) (Date)

……………………………… Witness (if any)	……………………………… Researcher
……………………………… (Signature)	……………………………… (Signature)
……………………………… (IC Number)	……………………………… (IC Number)
…………………………… (Date)	……………………………… (Date)
